# Alternative splicing of MALT1 controls signalling and activation of CD4^+^ T cells

**DOI:** 10.1038/ncomms11292

**Published:** 2016-04-12

**Authors:** Isabel Meininger, Richard A. Griesbach, Desheng Hu, Torben Gehring, Thomas Seeholzer, Arianna Bertossi, Jan Kranich, Andrea Oeckinghaus, Andrea C. Eitelhuber, Ute Greczmiel, Andreas Gewies, Marc Schmidt-Supprian, Jürgen Ruland, Thomas Brocker, Vigo Heissmeyer, Florian Heyd, Daniel Krappmann

**Affiliations:** 1 Research Unit Cellular Signal Integration, Institute of Molecular Toxicology and Pharmacology, Helmholtz Zentrum München—German Research Center for Environmental Health, Ingolstädter Landstrasse 1, 85764 Neuherberg, Germany; 2 Natural and Medical Sciences Institute at the University of Tübingen, Markwiesenstrasse 55, 72770 Reutlingen, Germany; 3 Research Unit Molecular Immune Regulation, Helmholtz Zentrum München—German Research Center for Environmental Health, Marchioninistrsse 25, 81377 München, Germany; 4 Institute for Immunology, Biomedical Center Munich, LMU Munich, Grosshaderner Strsse 9, 82152 Martinsried, Germany; 5 Institut für Klinische Chemie und Pathobiochemie, Klinikum rechts der Isar, Technische Universität München, 81675 München, Germany; 6 German Cancer Consortium (DKTK) and German Cancer Research Center (DKFZ), 69120 Heidelberg, Germany; 7 Molecular Immunopathology & Signal Transduction, III. Medizinische Klinik, Klinikum rechts der Isar, Technische Universität München, 81675 München, Germany; 8 German Center for Infection Research (DZIF), partner site Munich, 81675 München, Germany; 9 Free University Berlin, Institute of Chemistry and Biochemistry, RNA Biochemistry, Takustrasse 6, 14195 Berlin, Germany

## Abstract

MALT1 channels proximal T-cell receptor (TCR) signalling to downstream signalling pathways. With MALT1A and MALT1B two conserved splice variants exist and we demonstrate here that MALT1 alternative splicing supports optimal T-cell activation. Inclusion of exon7 in MALT1A facilitates the recruitment of TRAF6, which augments MALT1 scaffolding function, but not protease activity. Naive CD4^+^ T cells express almost exclusively MALT1B and MALT1A expression is induced by TCR stimulation. We identify hnRNP U as a suppressor of exon7 inclusion. Whereas selective depletion of MALT1A impairs T-cell signalling and activation, downregulation of hnRNP U enhances MALT1A expression and T-cell activation. Thus, TCR-induced alternative splicing augments MALT1 scaffolding to enhance downstream signalling and to promote optimal T-cell activation.

## Abstract (web summary)

MALT1 regulates NFκB signalling both as a scaffolding protein and as a protease. Here the authors show that during T cell activation the expression of MALT1 gene switches to an alternatively spliced variant, which increases TCR signal transduction due to enhanced TRAF6 binding.

## Graphical abstract

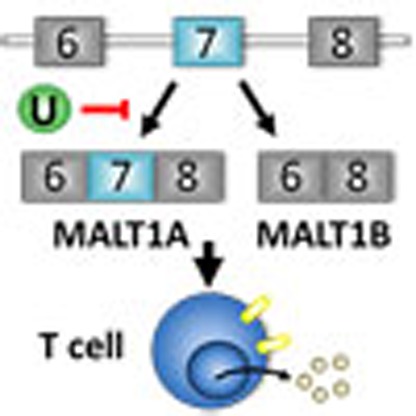

Antigenic stimulation of the T-cell receptor (TCR) together with a CD28 co-stimulatory receptor induces productive activation of naive CD4^+^ T cells. MALT1 (mucosa-associated lymphoid tissue protein 1) bridges TCR/CD28 co-engagement to cellular downstream signalling pathways to promote T-cell activation and effector functions^1^,^2^. As part of the CARMA1–BCL10–MALT1 (CBM) signalling complex, MALT1 channels upstream TCR signalling to the canonical IκB kinase (IKK)/nuclear factor-κB (NF-κB) signalling pathway. Three TRAF6-binding sites have been mapped on MALT1 (refs 3, 4). MALT1 recruits TRAF6 to the CBM complex to promote MALT1 ubiquitination and to facilitate activation of the IKK complex^5^. Besides its scaffolding function, MALT1 contains a paracaspase domain, and MALT1 proteolytic activity is induced on antigen stimulation in T cells^6^,^7^. MALT1 proteolytic activity is not directly involved in controlling canonical NF-κB signalling^7^,^8^. However, MALT1 cleavage of the deubiquitinases A20 and CYLD, the E3 ligase HOIL, the non-canonical NF-κB family member RelB or the RNA regulators Regnase-1 and Roquin have been associated with various functions for T-cell biology^6^,^7^,^9^,^10^,^11^,^12^,^13^.

Alternative splicing is a crucial and ubiquitous mechanism that controls gene expression at the co- and post-transcriptional level. In mammals, most pre-mRNAs are prone to alternative splicing, which results in the generation of multiple transcripts and proteins with diverse functions. Extensive changes in splicing patterns have been shown to occur in the immune response and especially in antigen-dependent T-cell activation^14^. Alternative splicing can act on multiple layers ranging from cell surface receptors, cytokines, signalling proteins to transcription factors, and thereby constitutes an essential regulatory mechanism for T-cell function^15^,^16^. A well-studied example is the TCR-induced exon exclusion of the transmembrane phosphatase CD45, which creates a negative-feedback regulation that counteracts T-cell activation^17^,^18^. However, in T cells, little is known how alternative splicing modulates expression and activity of intracellular signalling mediators and how this can influence T-cell signalling and activation.

Two conserved alternative splice isoforms of MALT1 have been assigned that differ only by inclusion (MALT1A) or exclusion (MALT1B) of exon7 that codes for 11 amino acids (aa 309–319 of human MALT1). However, neither expression nor functions of the two MALT1 alternative splice variants have been investigated. Here we identify heterogeneous nuclear ribonucleoprotein U (hnRNP U; SAF-A/SP120) as a factor that controls alternative MALT1 splicing and demonstrate that TCR-induced splicing of MALT1 increases relative MALT1A expression, which augments MALT1 scaffolding function and fosters activation of CD4^+^T cells.

## Results

### MALT1 exon7 supports optimal T-cell signalling and activation

**Figure 1:**
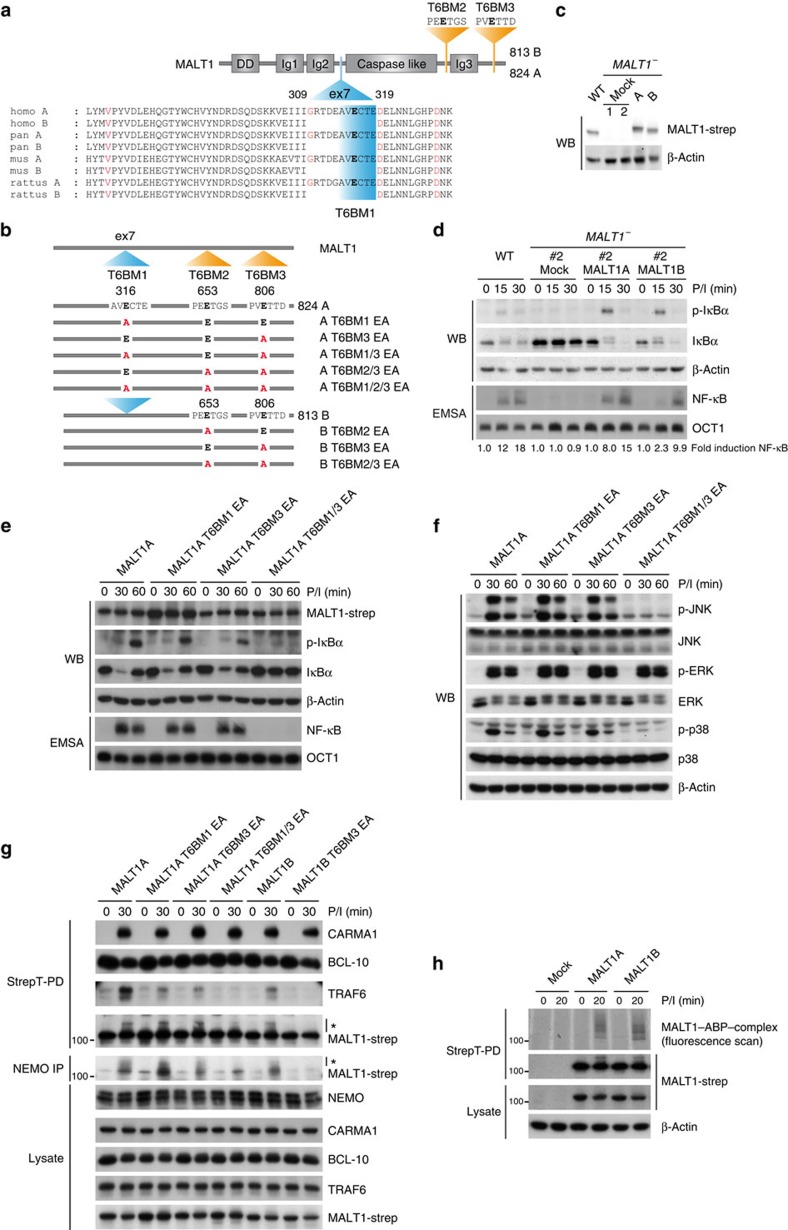
Conserved MALT1 exon7 enhances TRAF6 recruitment and NF-κB activation but not MALT1 activity. (**a**) Domain structure of MALT1 isoforms with different TRAF6-binding motifs (T6BMs) highlighted in orange and blue. Sequence conservation of T6BM1 in exon7 in different species is shown below. Protein domains are denoted by black boxes. DD, death domain, Ig, Immunoglobulin-like domain. (**b**) Schematics of the T6BMs in MALT1A and MALT1B. Different TRAF6-binding mutants were generated by glutamate (E) to alanine point mutations (A) as indicated. (**c**–**h**) MALT1-deficient Jurkat T-cell clone was reconstituted with StrepTagII (mock) or MALT1-StrepTagII variants. (**c**) MALT1 expression was checked by western blot (WB). (**d**) Reconstituted cells were stimulated with P/I for the indicated time points. NF-κB signalling was analysed by electrophoretic mobility shift assay (EMSA) and WB, and NF-κB signal was quantified relative to OCT1 control. (**e**,**f**) Cells transduced with MALT1A wild-type or MALT1A mutants were stimulated with P/I for the indicated time points. NF-κB and MAPK signalling were analysed by WB and EMSA. (**g**) CBM complex formation as well as TRAF6 recruitment were investigated by StrepT-PD after 30 min P/I stimulation. Binding of MALT1 to NEMO was monitored after NEMO IP. Modified MALT1 indicative of ubiquitination is marked by asterisk (*). (**h**) Proteins were precipitated by StrepT-PD after 20 min P/I stimulation and active MALT1 was detected using fluorescent MALT1-ABP probe. Data are representative of at least three independent experiments.

A comparison of mammalian transcriptome databases revealed that MALT1 is expressed in two alternative splice isoforms (Fig. 1a). The mRNA of the splice variants MALT1A (824 aa) and MALT1B (813 aa) only differs in the inclusion or exclusion of the 33-bp long exon7, which codes for amino acids 309–319 positioned between the Ig2- and caspase-like domains of human MALT1. The region was shown to contain a putative TRAF6-binding motif^4^. Expression of both splice variants, exon/intron boundaries, amino-acid sequences and TRAF6-binding site in MALT1 exon7 are highly conserved in mammals (Fig. 1a). This evolutionary and structural conservation points to a functional relevance of preserving the expression of the two MALT1 variants.

Two functional TRAF6-binding motifs (T6BM2 and T6BM3) have been identified in the C terminus of MALT1 (ref. 3; Fig. 1b). TRAF6 binding to T6BM1 within exon7 was demonstrated, but the role for T-cell signalling has not been investigated^4^. To analyse the putative isoform-specific effects on T-cell signalling, we generated MALT1-deficient Jurkat T cells using CRISPR/Cas9 for viral reconstitution (Supplementary Fig. 1a). The absence of MALT1 protein was verified by western blotting (Supplementary Fig. 1b). As expected, NF-κB activation in response to PMA/Ionomycin (P/I), but not tumour necrosis factor-α (TNF-α) stimulation, was lost in Jurkat T-cell clone #2 that lacks MALT1 (Supplementary Fig. 1c). To analyse the differential effects of MALT1 isoforms, MALT1-deficient Jurkat T cells were lentivirally reconstituted with MALT1A or MALT1B. Both the isoforms were expressed at equivalent levels and in the range of endogenous MALT1 from Jurkat T cells (Fig. 1c). Reconstitution with either MALT1A or MALT1B rescued NF-κB signalling, but the activation of IκBα degradation and NF-κB in MALT1A-expressing cells was more rapid and slightly stronger when compared with MALT1B cells (Fig. 1d), indicating that inclusion of exon7 can facilitate TRAF6 recruitment to promote optimal NF-κB activation.

We wanted to validate that the putative binding motif in MALT1 exon7 is crucial for signalling in Jurkat T cells. For this, we reconstituted MALT1-deficient Jurkat T cells with MALT1A or MALT1B constructs mutated in one or more TRAF6-binding sites (Fig. 1b; Supplementary Fig. 1d). Whereas mutation of the TRAF6-binding motif 2 (T6BM2) next to the Ig3 in MALT1B does not affect signalling, activation of NF-κB, JNK and p38 completely relies on the presence of the most C-terminal T6BM3 (Supplementary Fig. 1e,f). In contrast, only the combined mutation of T6BM1 in exon7 and the C-terminal T6BM3 (T6BM1/3 EA) in MALT1A abrogates the activation of downstream signalling pathways (Fig. 1e,f). To prove that the mutations in individual or multiple T6BMs affect TRAF6 recruitment, we performed StrepT-PD of transduced Jurkat T cells (Fig. 1g). Constitutive BCL10 association and inducible CARMA1 recruitment were not affected by MALT1 exon7 inclusion or mutations of TRAF6 sites, but TRAF6 recruitment was diminished in MALT1B compared with MALT1A. In line with the effects on signalling, mutation of the binding site T6BM3 in MALT1B alone and the combined mutation of T6BM1/3 in MALT1A abolished TRAF6 recruitment. Also, MALT1 ubiquitin modification was severely reduced only in the absences of any functional TRAF6-binding site, which prevented recruitment of ubiquitinated MALT1 to the IKK regulatory subunit NEMO (Fig. 1g)^5^. Thus, the mutagenesis of individual or multiple TRAF6-binding sites confirms that exon7 is significantly contributing to the activation of downstream signalling pathways.

To test whether splicing affects MALT1 proteolytic activity in response to T-cell stimulation, we measured MALT1 protease activation in reconstituted Jurkat T cells using fluorescently labelled MALT1 activity-based probe (MALT1-ABP; Fig. 1h)^19^. As expected, MALT1 activation was detected on P/I stimulation and active MALT1 was heavily modified, which most likely corresponds to previously described active MALT1 ubiquitin conjugates^20^. However, MALT1A and MALT1B were activated to a similar extent and also cleavage of the MALT1 substrate CYLD was not altered in MALT1A- or MALT1B-expressing cells (Supplementary Fig. 1g), revealing that MALT1 protease activation is not affected by inclusion or exclusion of exon7.

**Figure 2:**
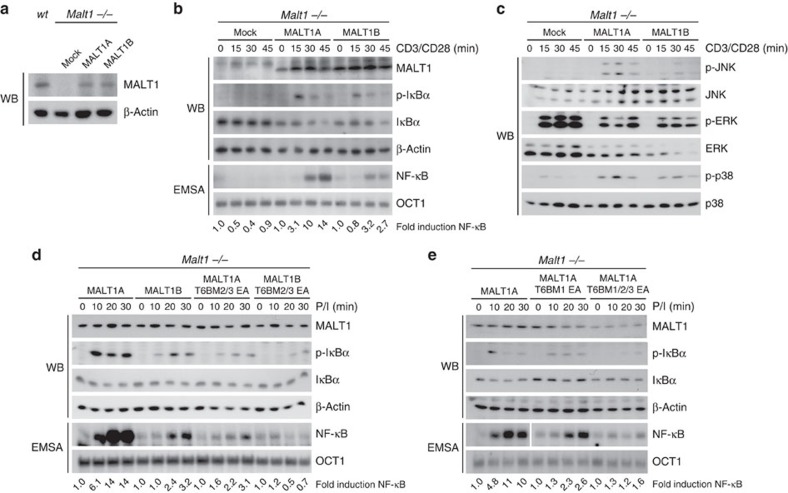
MALT1A promotes stronger NF-κB and JNK activation on T-cell stimulation. (**a**–**c**) CD4^+^ T cells from MALT1-deficient mice (C57BL/6J) were reconstituted with mock, MALT1A or MALT1B. (**a**) MALT1 expression levels of T cells from *wt* mice or reconstituted *Malt1*^−/−^ mice were determined by western blot (WB). NF-κB (**b**) and MAPK signalling (**c**) were analysed by WB after stimulation with anti-CD3/CD28 as indicated. NF-κB DNA binding was determined by electrophoretic mobility shift assay (EMSA). NF-κB signal was quantified relative to OCT1 control. (**d**,**e**) MALT1-deficient mice (C57BL/6J) were reconstituted with MALT1A, MALT1B or different TRAF6-binding mutants as indicated (Fig. 1b). IκBα phosphorylation and degradation were analysed by WB after P/I stimulation and DNA binding of NF-κB was monitored by EMSA. NF-κB signal was quantified relative to OCT1 control. Data are representative of at least three independent experiments.

To test whether the MALT1 splice variants exert differential effects on CBM downstream signalling in primary T cells, we purified CD4^+^ T cells from *Malt1*^−/−^ mice and reconstituted the cells by retroviral transduction with either MALT1A or MALT1B. Reconstituted T cells were expanded and transduced cells were purified by the co-expressed surface marker Thy1.1 yielding 80–90% Thy1.1-positive T cells (Supplementary Fig. 2a). As expected, the absence of MALT1 completely abolished NF-κB and JNK signalling on T-cell stimulation (Supplementary Fig. 2b)^1^. Expression of MALT1A and MALT1B was comparable to endogenous MALT1 levels in T cells from *wt* mice (Fig. 2a). To determine induction of NF-κB and MAPK signalling, reconstituted T cells were restimulated with anti-CD3/CD28 or P/I (Fig. 2b,c; Supplementary Fig. 2c,d). Expression of both the MALT1 isoforms rescued NF-κB signalling on anti-CD3/CD28 or P/I stimulation. Congruent with our observations in MALT1-deficient Jurkat T cells, IκBα degradation was more rapid and NF-κB activation was stronger in cells expressing MALT1A when compared with MALT1B. Also, JNK phosphorylation was rescued on MALT1 expression and MALT1A was more potent in mediating JNK activation than MALT1B after anti-CD3/CD28 or P/I stimulation. MALT1 was also able to rescue the partial defect in p38 activation that has been observed earlier in MALT1-deficient T cells^1^, but p38 activation did not rely on the MALT1 isoforms. Moreover, despite a reduction in ERK amounts in MALT1A- or MALT1B-expressing cells, no differences in the extent of inducible ERK phosphorylation were detected using the two splice variants.

To investigate whether the TRAF6-binding motifs in the C terminus and exon7 contribute to differential effects on MALT1 downstream signalling in primary T cells, we performed reconstitutions using a series of MALT1 point mutations to destroy T6BMs individually or in combination (Fig. 1b) and determined the effects on NF-κB signalling in primary T cells. Mutation of the C-terminal TRAF6 motifs (T6BM2/3 EA) in MALT1A led to a similar reduction in P/I-triggered NF-κB signalling activation as exclusion of exon7 in MALT1B (Fig. 2d). Moreover, NF-κB signalling was decreased on destruction of the T6BM1 in exon7 MALT1A (T6BM1 EA; Fig. 2e). Only the combined destruction of T6BMs in either MALT1A (T6BM1/2/3 EA) or MALT1B (T6BM2/3 EA) completely abolished the NF-κB response (Fig. 2d,e). Thus, in primary T cells, intact TRAF6-binding sites in the C terminus of MALT1B are sufficient to induce moderate downstream signalling, but cooperation between C-terminal and exon7-encoded TRAF6-binding motifs promote optimal NF-κB signalling.

**Figure 3:**
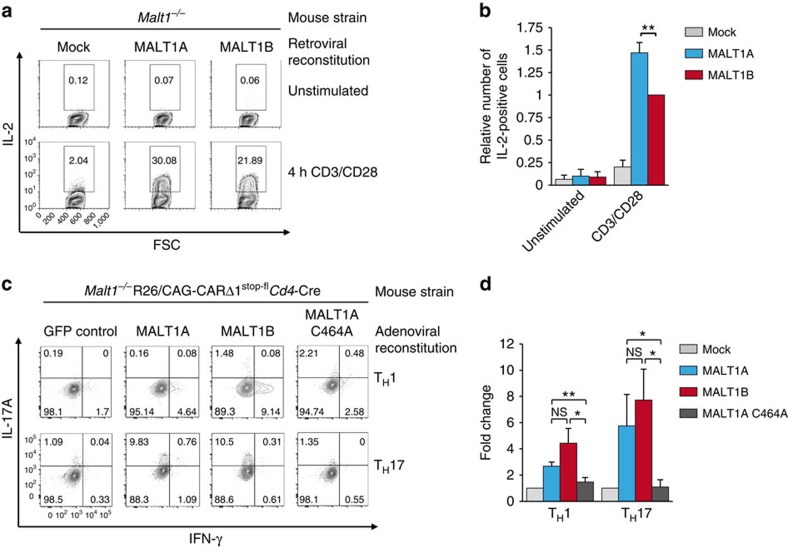
MALT1A expression enhances IL-2 production but not T_H_17 differentiation in CD4^+^ T cells. (**a**,**b**) *Malt1*^−/−^ CD4^+^ T cells were retrovirally reconstituted with either mock, MALT1A or MALT1B. (**a**) Reconstituted T cells were stimulated with anti-CD3/CD28 for 4 h and intracellular IL-2 production was analysed by FACS. (**b**) Quantification of IL-2 production. The percentage of IL-2-positive cells from MALT1B-expressing cells was set to 1. Depicted is the mean±s.d. (*n*=3) (**c**,**d**) CD4^+^ T cells from *Malt1*^−/−^R26*/*CAG-CARΔ1^stop-fl^*Cd4*-Cre mice were adenovirally reconstituted with GFP control, MALT1A, MALT1B or MALT1A C464A. Cells were cultured for 3 days under T_H_1- or T_H_17-polarizing conditions and restimulated with P/I. GFP^+^ cells were analysed for intracellular IFN-γ and IL-17A levels by FACS and numbers in quadrants represent percentage of IFN-γ- or IL-17A-positive cells. (**d**) Data show mean±s.d. (*n*=4). *P<0.05; **P<0.01; NS, not significant; unpaired *t*-test.

In Jurkat T cells, we observed the effects of exon7 on downstream signalling, but not on paracaspase activity, suggesting that inclusion of exon7 is primarily modulating MALT1 scaffolding function. Interleukin (IL)-2 expression critically depends on MALT1 scaffolding and partially on MALT1 cleavage activity^5^,^8^,^21^,^22^,^23^. Indeed, IL-2 production after anti-CD3/CD28 stimulation was rescued in T cells retrovirally reconstituted with MALT1A or MALT1B, but the number of IL-2-producing cells was increased in MALT1A transduced cells (Fig. 3a,b). Therefore, the stronger potency of MALT1A to promote downstream signalling correlates with a more robust IL-2 production. T_H_17 differentiation was completely abolished in MALT1 protease defective mice and is thus apparently independent of MALT1 scaffolding function^22^,^24^. To investigate whether inclusion of exon7 influences T_H_17 differentiation, we adenovirally transduced naive CD4^+^ T cells expressing a signalling-deficient coxsackie adenovirus receptor (CARΔ1) from *Malt1*^−/−^R26*/*CAG-CARΔ1^stop-fl^*Cd4*-Cre mice^12^,^25^ using green fluorescent protein (GFP) as infection marker (Supplementary Fig. 3a). We optimized *ex vivo* differentiation under T_H_1- or T_H_17-inducing conditions and found that T_H_17 differentiation was absent in *Malt1*^−/−^ T cells in the presence of anti-CD3 and irradiated antigen-presenting cells (APCs; Supplementary Fig. 3b). Interestingly, also T_H_1 differentiation was diminished in the absence of MALT1, indicating that depending on the conditions MALT1 does not only control T_H_17 differentiation^24^. Using these conditions, we monitored the number of IFNγ-producing T_H_1 cells and IL-17A-expressing T_H_17 cells in MALT1-deficient cells after viral rescue (Fig. 3c,d). Both the MALT1 splice variants induced T_H_17 and T_H_1 differentiation to a similar extend. In contrast, the catalytically inactive MALT1A C464A mutant was unable to promote T_H_1 and T_H_17 differentiation. Thus, the biochemical and functional data provide evidence that alternative MALT1 splicing modulates MALT1 scaffolding and downstream signalling, but does not affect MALT1 protease activity and cell fate decisions.

### TCR signalling upregulates MALT1A in CD4^+^ T cells

**Figure 4:**
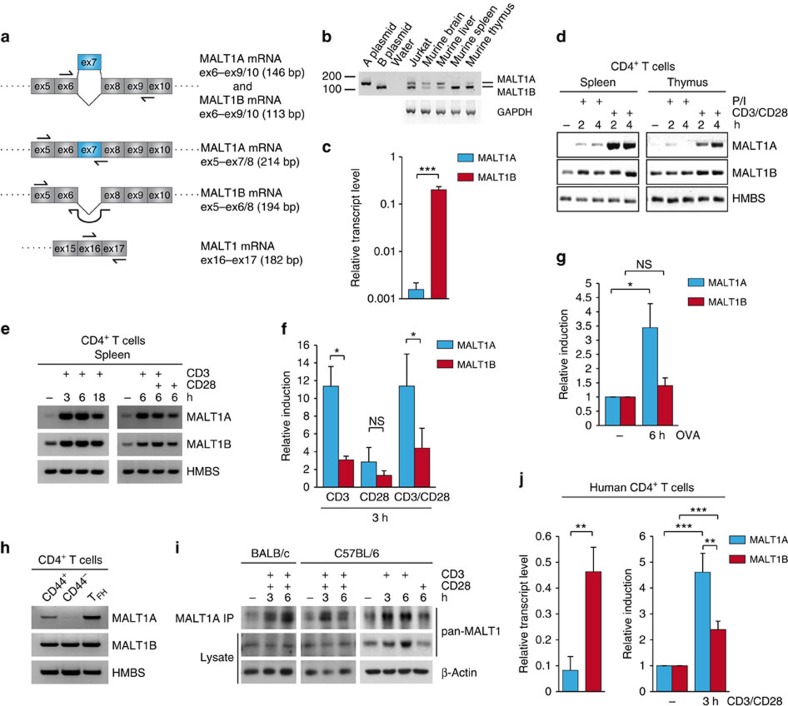
TCR ligation induces MALT1 splicing and exon7 inclusion in CD4^+^ T cells. (**a**) Scheme of MALT1 primers amplifying MALT1A (ex5–ex7/8), MALT1B (ex5–ex6/8) or MALT1A/B (ex6–ex9/10 or ex16–ex17). (**b**) Analysis of MALT1 mRNA levels by RT–PCR in Jurkat T cells and different murine tissues from BALB/c mice using ex6–ex9/10 primers amplifying both isoforms. GAPDH served as control. (**c**–**f**) MALT1A and MALT1B mRNA levels in CD4^+^ T cells from BALB/c mice were investigated by qPCR (**c**,**f**) or semi-qPCR (**d**,**e**) using isoform-specific ex5–ex7/8 or ex5–ex6/8 primers. Transcript levels were normalized to Hydroxymethylbilane Synthase (HMBS) mRNA levels. For semi-qPCR, amplification cycles were adjusted for MALT1A (35 cycles) and MALT1B (28 cycles). (**g**) MALT1A and MALT1B mRNA levels were analysed in OT-II CD4^+^ T cells cultured for 6 h alone or with irradiated wild-type APCs unloaded or loaded with 1 μg ml^−1^ OVA peptide. HMBS served as internal control and relative induction was determined comparative to unloaded APCs cultured with OT-II T cells. (**h**) Activated CD62L^−^CD44^+^ effector memory T cells, naive CD62L^+^CD44^−^ T cells and CXCR5^+^PD1^+^ T-follicular helper (T_FH_) cells were sorted from immunized BALB/c mice. RNA was isolated, and MALT1A and MALT1B levels were analysed by semi-qPCR using isoform-specific primer pairs. (**i**) CD4^+^ T cells from BALB/c and C57BL/6J mice were treated with anti-CD3, anti-CD28 or both stimuli. After lysis, MALT1A was precipitated using anti-MALT1A antibody. A pan-MALT1 antibody was used for detection by western blot (WB). (**j**) Primary human CD4^+^ T cells from four donors were stimulated with anti-CD3/CD28 for 3 h. MALT1A and MALT1B mRNA levels were measured by qPCR using human MALT1A- or MALT1B-specific primers. RP2 served as internal control and relative induction was determined comparative to unstimulated cells. Data are representative for two (**b**,**d**) or three (**c**,**e**–**i**) or four (**j**) independent experiments. Depicted is the mean±s.d. (**c**,**e**–**i**; *n*=3) or (**j**; *n*=4). *P<0.05; **P<0.01; ***P<0.001; NS, not significant; unpaired *t*-test.

To elucidate whether the differences in MALT1 signalling strength observed on inclusion or skipping of exon7 are relevant for activation of CD4^+^ T cells, we next investigated the expression of MALT1 splice variants on RNA level. We designed primer pairs that either flank exon7 (ex6–ex9/10) to amplify MALT1A/B simultaneously or exon spanning primers that selectively amplify either MALT1A (ex5–ex7/8) or MALT1B (ex5–ex6/8; Fig. 4a,b; Supplementary Fig. 4a). Semi-quantitative PCR (qPCR) demonstrated that MALT1A and MALT1B are expressed at equivalent levels in Jurkat T cells, but differentially expressed in murine tissues (Fig. 4b). Whereas MALT1A and MALT1B were present in murine brain and liver, the lymphoid organs spleen and thymus contained almost exclusively the shorter MALT1B variant, indicating that MALT1 is prone to alternative splicing in different cells and tissues. To better compare MALT1 isoform expression, we amplified either MALT1A or MALT1B by qPCR. In murine CD4^+^ T cells, MALT1B transcripts were more abundant and expressed ∼100-fold more than MALT1A mRNA, which was hardly detectable (Fig. 4c). Next, we analysed the changes in mRNA expression of the two MALT1 isoforms after TCR/CD28 stimulation by semi-qPCR or qPCR (Fig. 4d–f). Again, MALT1A transcripts in CD4^+^ T cells purified from spleen or thymus were almost undetectable, but MALT1A was induced by a factor of 10–12 on anti-CD3/CD28 or anti-CD3 stimulation. P/I stimulation was not sufficient to induce MALT1A, suggesting that proximal TCR signalling events are critical. MALT1B levels were only moderately induced (2–4-fold) on anti-CD3 or anti-CD3/CD28 ligation, which was also seen for overall MALT1 expression (Supplementary Fig. 4b). Induction of MALT1A, but not MALT1B expression, was also observed on stimulation of primary OT-II T cells with the ovalbumin (OVA) peptide-loaded APCs (Fig. 4g). Thus, TCR ligation induces alternative MALT1 splicing and inclusion of exon7. Despite the higher expression of MALT1B, TCR signalling triggers a relative increase of MALT1A transcript levels in CD4^+^ T cells.

To confirm that the induction of MALT1A was also seen *in vivo*, BALB/c mice were immunized by intravenous injection of sheep erythrocytes. After 7 days, naive T cells (CD62L^+^CD44^−^), activated effector memory T cells (CD62L^-^CD44^+^) and T-follicular helper cells (T_FH_; CXCR5^+^PD1^+^) were purified. Whereas MALT1B transcripts were not changed in the three T-cell subsets, the MALT1A splice variant was highly expressed in activated T cells and T_FH_ cells, but low in naive T cells (Fig. 4h). Thus, alternative MALT1 splicing is also induced *in vivo*, leading to a MALT1A upregulation in activated CD4^+^ T-cell subsets.

To address whether TCR-induced alternative splicing and exon7 inclusion also enhanced MALT1A protein amounts, we generated a rabbit polyclonal antibody against exon7 (aa 309–316), which is identical in human and murine MALT1A. On overexpression of MALT1, the antibody recognized exclusively Flag-MALT1A in western blots or after immunoprecipitation (IP) and did not cross-react with Flag-MALT1B (Supplementary Fig. 4c). To detect endogenous MALT1A, we had to enrich MALT1A by IP and used the higher affinity pan-MALT1 antibody for immunoblot detection (Fig. 4i). MALT1A protein was hardly detectable in CD4^+^ T cells, but anti-CD3/CD28 stimulation induced MALT1A expression in T cells from different mouse strains. Again, anti-CD3 stimulation alone was able to induce MALT1A expression. Owing to the high MALT1B expression levels, total MALT1 protein expression was largely unchanged after stimulation and we only observed a modest upregulation in C57BL/6 mice. Taken together, the results demonstrate that TCR ligation leads to an alteration in the MALT1 isoform ratio and an increase in the relative amounts of MALT1A in activated T cells.

Since MALT1 exon7 is conserved in mammals, we asked whether differences in MALT1A and MALT1B levels are also seen in human T cells purified from peripheral blood. Human CD4^+^ T cells expressed more MALT1B mRNA compared with MALT1A (Fig. 4j). Again, anti-CD3/CD28 stimulation induced a relative increase in MALT1A expression. Even though the effects were not as pronounced as in splenic murine CD4^+^ T cells, the data reveal that MALT1 is prone to alternative splicing in human T cells.

### hnRNP U negatively regulates inclusion of MALT1 exon7

**Figure 5:**
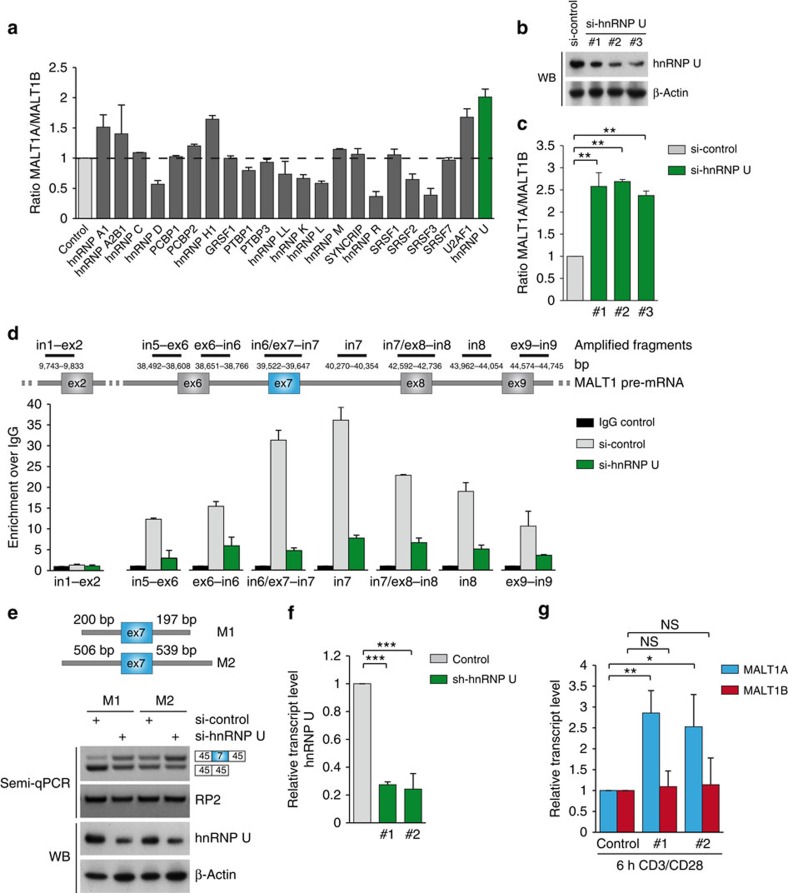
Splice factor hnRNP U counteracts exon7 inclusion and MALT1A expression in T cells. (**a**) Identification of RNA-binding proteins involved in MALT1 splicing by RNA interference. Jurkat T cells were transfected with smart pool siRNA against the depicted proteins and the ratio MALT1A/B mRNA was analysed by qPCR. (**b**) Knockdown of hnRNP U after transfection of three independent siRNAs in Jurkat T cells. (**c**) qPCR of MALT1A and MALT1B mRNA after downregulation of hnRNP U in Jurkat T cells. Shown is the MALT1A/B mRNA ratio. (**d**) Binding of hnRNP U to MALT1 pre-mRNA. IgG control IP or anti-hnRNP U IP was carried out from extracts of si-control- or si-hnRNP U-transfected Jurkat T cells. IP of MALT1 pre-mRNA was detected by qPCR using primers amplifying the depicted fragments. Primers amplifying MALT1 pre-mRNA in1–ex2 served as negative control. (**e**) Exon7 spanning minigenes M1 and M2 were transfected into Jurkat T cells together with si-control or si-hnRNP U. Transcript levels of the minigene with or without exon7 were analysed by RT–PCR. (**f**) hnRNP U knockdown on mRNA level in CD4^+^ T cells after transduction of either control or two hnRNP U adenoviruses. hnRNP U levels were analysed by qPCR using sorted GFP^+^ CD4^+^ T cells after 6 h anti-CD3/CD28 stimulation. (**g**) Analysis of MALT1A and MALT1B transcript levels after 6 h anti-CD3/CD28 stimulation of either control or sh-hnRNP U-transduced cells. All qPCR were performed using HMBS as internal control. Data are representative for two (**a**,**d**) or three (**b**,**c**,**e**–**g**) independent experiments. Depicted is the mean±s.d. (**a**,**d**; *n*=2) or (**c**,**f**,**g**; *n*=3). *P<0.05; **P<0.01; ***P<0.001; NS, not significant; unpaired *t*-test.

To corroborate our findings that MALT1 is prone to alternative splicing, we searched for RNA-binding proteins that influence the relative expression of MALT1A and MALT1B. Since both the MALT1 isoforms are expressed constitutively in Jurkat T cells (Fig. 4a), we initiated two independent small interfering RNA (siRNA) screens after transfection of smart pool siRNA against putative RNA-binding proteins that have been connected to splicing. We determined relative expression of MALT1A and MALT1B either by qPCR (Fig. 5a) or radioactive PCR (Supplementary Fig. 5a; Supplementary Table 1). In both data sets, we identified the splice factor hnRNP U as a candidate that induced the most robust inclusion of MALT1 exon7 and thus the strongest shift towards the MALT1A expression. Knockdown of hnRNP U by the smart pool siRNA was verified by qPCR and MALT1 exon7 inclusion was also evident by semi-qPCR (Supplementary Fig. 5b,c). Next, we confirmed the relative increase in the expression levels of MALT1A versus MALT1B using three independent siRNAs (Fig. 5b,c). Thus, RNA interference identified hnRNP U as a negative regulator of exon7 inclusion in Jurkat T cells.

To validate the impact of hnRNP U, we investigated binding of hnRNP U to the MALT1 pre-mRNA. RNA-binding protein IP (RIP) was performed with anti-hnRNP U or IgG control antibodies in Jurkat T cells and the MALT1 pre-mRNA near exon7 (between exon6 and exon9) or in a more distant region (exon2) was amplified by qPCR (Fig. 5d). The pre-mRNA of MALT1 in the region of exon7 was enriched on hnRNP U RIP and highest values were obtained for the fragments in proximity or covering exon7. Specificity was confirmed by hnRNP U knockdown and amplification of an in1–ex2 fragment, which was not enriched after hnRNP U RIP. hnRNP U association to MALT1 pre-mRNA was also seen in semi-qPCR using in6/ex7–in7 primers (Supplementary Fig. 5d). To address whether *cis*-regulatory sequences around exon7 are able to control skipping or inclusion, we constructed a minigene bearing exon7 and flanking intronic sequences cloned between constitutive exon3 and 7 of CD45. Whereas Jurkat T cells transfected with the longer construct M2 resulted in equivalent exon7 skipping/inclusion, exon7 was almost completely excluded using the shorter fragment M1 (Fig. 5e). hnRNP U induced exon7 exclusion in both minigenes confirming its role as a negative regulator of exon7 splicing. Thus, our data reveal that enhancer and silencer regions in the vicinity to exon7 control alternative MALT1 splicing and that binding of hnRNP U impairs exon7 inclusion.

To determine whether hnRNP U is also controlling MALT1A transcript levels on TCR stimulation in primary T cells, we used adenoviral small hairpin RNA (shRNA) knockdown in murine CD4^+^ T cells. Infected CD4^+^ T cells were sorted based on co-expression of GFP and an efficient downregulation of hnRNP U was obtained with two independent shRNAs (Fig. 5f; Supplementary Fig. 5e). Indeed, increased MALT1A expression after anti-CD3 stimulation was detected after downregulation of hnRNP U (Fig. 5g). In contrast, MALT1B expression was not altered. We asked whether enhanced MALT1A expression might be correlated with a decrease in hnRNP U after T-cell activation. However, on RNA level, the MALT1A suppressor hnRNP U was induced, but at the same time other splicing factors that enhanced MALT1A in the screens (for example, hnRNP R, SRSF3 and SRSF9) were increased (Supplementary Fig. 5f). Thus, TCR-induced alternative splicing of exon7 and the relative expression of MALT1A/B transcripts in CD4^+^ T cells is regulated by hnRNP U, but alternative MALT1 splicing seems to be regulated by a complex network of negatively and positively acting splice factors.

### MALT1A supports and hnRNP U counteracts T-cell activation

**Figure 6:**
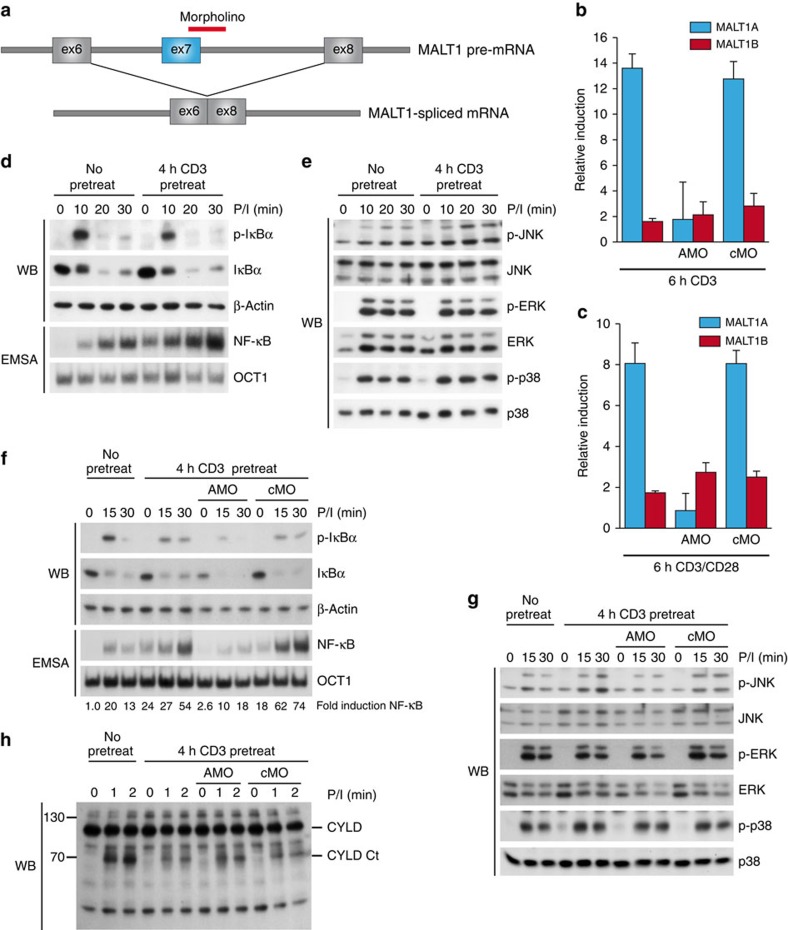
Induction of MALT1A augments T-cell signalling. (**a**) Scheme for vivo morpholino (MO)-induced disruption of MALT1A expression. MO is designed to bind the 3′-splice site of exon7/intron7 in MALT1 pre-mRNA to prevent spliceosomal recognition. (**b**,**c**) CD4^+^ T cells from BALB/c mice were treated with morpholino against MALT1A (AMO), control MO (cMO) or kept untreated for 18 h before anti-CD3 or anti-CD3/CD28 stimulation for 6 h. MALT1 mRNA levels were analysed by qPCR using isoform-specific primers. HMBS served as internal control and relative induction was determined by comparing stimulated to unstimulated cells. (**d**,**e**) CD4^+^ T cells from BALB/c mice were pretreated with anti-CD3 for 4 h and afterwards stimulated with P/I for the indicated times. Phosphorylation and degradation of IκBα (**d**) as well as MAPK activation (**e**) were analysed by WB. Electrophoretic mobility shift assay (EMSA) was used to detect DNA binding of NF-κB (**d**). (**f**–**h**) Untreated, AMO- or cMO-treated CD4^+^ T cells were pretreated with anti-CD3 (4 h) before stimulation with P/I. (**f**,**g**) NF-κB and MAPK signalling were analysed by EMSA and WB. NF-κB signal was quantified relative to OCT1 control. (**h**) CYLD cleavage was monitored by WB. Data are representative of two (**h**) or three independent experiments (**b**–**g**). In **b** and **c**, the mean±s.d. (*n*=3) is depicted.

To test a potential influence of alternative MALT1 splicing on cellular signalling in T cells, we designed a morpholino oligomer (MO) targeting the exon7–intron7 boundary of the MALT1 pre-mRNA to selectively prevent alternative inclusion of exon7 and thus MALT1A expression (Fig. 6a). MALT1A MO (AMO) suppressed MALT1A mRNA and protein expression in mouse embryonic fibroblasts (MEFs; Supplementary Fig. 6a,b). Also, in murine T cells, AMO treatment abolished anti-CD3- or anti-CD3/CD28-stimulated induction of MALT1A transcripts without affecting transcript levels of MALT1B (Fig. 6b,c; Supplementary Fig. 6c).

Since freshly isolated CD4^+^ T cells lack considerable MALT1A expression, no changes in NF-κB signalling and JNK activation were observed in AMO-treated cells stimulated with anti-CD3/CD28 or P/I (Supplementary Fig. 6d,e). To test whether induction of MALT1A on TCR ligation can augment T-cell signalling, we pretreated CD4^+^ T cells with anti-CD3 to induce MALT1A expression and restimulated the cells with P/I, taking advantage of the fact that P/I bypasses upstream TCR stimulation that may be altered due to the prestimulation. NF-κB was enhanced after 4 h TCR prestimulation, and the activation of NF-κB and JNK was augmented on secondary P/I restimulation, while the activities of ERK and p38 were unaffected in these experimental conditions (Fig. 6d,e). To test whether MALT1A induction is required to augment NF-κB and JNK signalling on restimulation, we cultured the purified CD4^+^ T cells in the presence of AMO during anti-CD3 pretreatment to prevent MALT1A induction (Fig. 6b). Again, TCR stimulation augmented NF-κB activation and slightly enhanced JNK phosphorylation on secondary P/I stimulation in untreated or in cMO-treated T cells (Fig. 6f,g). In contrast, inhibition of MALT1A by AMO severely reduced NF-κB activation and mildly impaired JNK signalling. Both the pathways are activated after AMO treatment to a similar degree as in CD4^+^ T cells without prestimulation. Again, ERK and p38 activation were not affected, revealing that MALT1A induction is selectively affecting NF-κB and JNK signalling. Since JNK activation in Jurkat T cells has been linked to CYLD cleavage by MALT1 paracaspase^10^, we checked for changes in MALT1 paracaspase activity and CYLD cleavage in primary T cells depending on the induction of MALT1A (Fig. 6h; Supplementary Fig. 6f). Inhibition of MALT1A induction did not alter MALT1 protease activation. CYLD cleavage was reduced after anti-CD3 pretreatment, but morpholino treatment did not alter CYLD cleavage after P/I restimulation, revealing that augmented JNK signalling is not caused by enhanced MALT1 activity.

**Figure 7:**
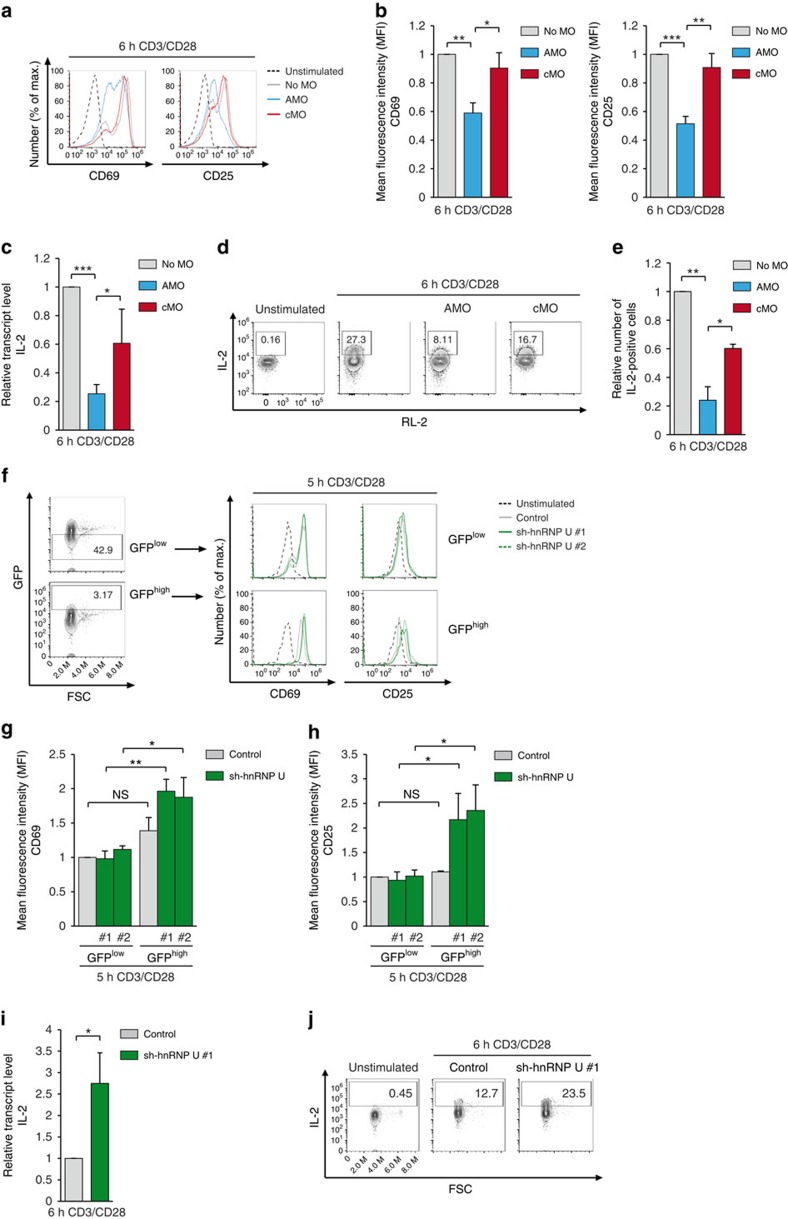
MALT1A induction and hnRNP U downregulation augment T-cell activation. (**a**–**e**) CD4^+^ T cells from BALB/c mice were left untreated or were treated with AMO or cMO before stimulation. (**a**) Cell surface expression of CD69 and CD25 was determined by FACS after 6 h of anti-CD3/CD28 stimulation. (**b**) Mean fluorescence intensity (MFI) values of CD25 and CD69 after stimulation of MO-treated cells. (**c**) Induction of IL-2 mRNA in CD4^+^ T cell (no MO, AMO or cMO treated) after anti-CD3/CD28 stimulation was determined by qPCR. (**d**) Intracellular IL-2 staining after anti-CD3/CD28 and different MO treatments was analysed by FACS. (**e**) Number of IL-2-positive cells as gated in **d** was quantified in relation to stimulated control cells (no MO). (**f**–**j**) CD4^+^ T cells from R26*/*CAG-CARΔ1^stop-fl^*Cd4*-Cre mice were transduced with control or sh-hnRNP U adenoviruses and stimulated with anti-CD3/CD28. (**f**) Cells gated for GFP^low^ and GFP^high^ expression (left) were analysed for CD25 and CD69 surface expression (right). (**g**,**h**) CD25 and CD69 MFI values after stimulation of adenoviral-transduced cells. MFI was directly compared between GFP^low^ (no hnRNP U knockdown)- and GFP^high^ (hnRNP U knockdown)-expressing cells. (**i**) Relative IL-2 mRNA levels in sorted GFP^+^ CD4^+^ T cell on adenoviral transduction of control or sh-hnRNP U #1 after anti-CD3/CD28 stimulation was determined by qPCR. (**j**) Intracellular IL-2 levels after anti-CD3/CD28 stimulation in sorted GFP^+^ cells was analysed by FACS. Histograms and dot blots show representatives of two (**j**) or three (**a**–**i**) independent experiments. (**b**,**c**,**e**,**g**–**i**) Depicted is the mean±s.d. (*n*=3). *P<0.05; **P<0.01; ***P<0.001; NS, not significant; unpaired *t*-test.

In *Malt1*^−/−^ T cells, expression of MALT1A promoted a more robust T-cell signalling and IL-2 production when compared with MALT1B (Figs 2 and 3). To test whether MALT1A induction is also required for robust T-cell activation, AMO- and cMO-treated CD4^+^ T cells were stimulated with anti-CD3/CD28 for 6 h and surface expression of activation markers CD25 and CD69 as well as induction of IL-2 were examined. Indeed, prevention of MALT1A induction led to reduced surface expression of CD69 and CD25 (Fig. 7a,b). Further, induction of IL-2 mRNA as well as the number of IL-2-expressing cells were decreased in AMO-treated CD4^+^ T cells (Fig. 7c-e), reflecting that TCR-triggered induction of MALT1A is necessary to promote optimal T-cell activation.

To provide evidence that alternative splicing and MALT1A induction is involved in augmented T-cell activation, we determined the effects of knocking down the splicing factor hnRNP U that counteracts inducible MALT1A production in CD4^+^ T cells (Fig. 5g). Indeed, CD25 surface expression was significantly enhanced in GFP^+^ cells containing the adenoviral hnRNP U shRNA, but not in the GFP^−^ cells (Fig. 7f–h). The weaker differences in CD69 upregulation after hnRNP U knockdown is most likely due to the rapid localization of intracellular CD69 at the cell surface on T-cell activation^26^. We sorted GFP expressing CD4^+^ T cells to determine IL-2 expression on mRNA and protein level. Again, sh-hnRNP U augmented IL-2 induction and enhanced the number of IL-2-expressing cells (Fig. 7i,j). Taken together, prevention or enhancement of MALT1A induction by morpholinos or sh-hnRNP U, respectively, exerted opposing effects on T cell activation, suggesting that MALT1 alternative splicing controls relative MALT1A and MALT1B levels and thus functions as a molecular switch to tune T-cell activation.

## Discussion

MALT1 paracaspase controls adaptive and innate immune signalling pathways and is a key regulator of T-cell activation^27^. Despite the fact that two conserved MALT1 splice variants exist in mammals neither expression nor the functional relevance of these MALT1 isoforms has been explored. Here we show that MALT1 is prone to alternative splicing. While murine CD4^+^ T cells express almost exclusively the shorter isoform MALT1B, the longer exon7 containing variant MALT1A is induced on TCR stimulation. Lower MALT1A expression and induction of MALT1A after T-cell stimulation was also observed in human CD4^+^ T cells from peripheral blood. We find differences in the levels of MALT1A/B in human and murine cells, but the different sources, purification protocols and stimulatory conditions preclude a quantitative comparison. We identified the RNA-binding protein hnRNP U as a negative regulator of exon7 inclusion in human Jurkat and murine primary T cells and we show that MALT1A upregulation enhances T-cell signalling and activation. Despite the induction of MALT1A, MALT1B remained the more abundant isoform, suggesting that not the absolute amounts, but the shifted balance in MALT1A versus MALT1B expression is defining downstream effects. The relevance of MALT1 alternative splicing is also underscored by the observation that MALT1A transcripts are elevated *in vivo* in T cells with a more activated phenotype such as effector memory T cells and T_FH_ cells.

Alternative splicing is an important mechanism regulating cell function during the immune response^14^. Global analysis of splicing events using exon array demonstrated extensive alternative exon usage in many immune regulators during T-cell activation^28^. Alternative exon7 usage of MALT1 in T cells was never reported using exon arrays, most likely because the short exon7 cannot be detected in global analyses. TCR-induced inclusion of exon7 generates the hyperactive MALT1A isoform that provides an additional and functional TRAF6-binding motif that boosts NF-κB activation and slightly enhances JNK signalling in primary T cells. Also, in Jurkat T cells MALT1A was slightly more effective in triggering downstream signalling, but interestingly deletion of exon7 in MALT1B exerted a more severe effect than point mutation of T6BM1, suggesting that the role of exon7 may not be restricted to TRAF6 recruitment. Further, whereas in Jurkat T cells one of two T6BMs was largely sufficient to trigger full-NF-κB signalling, decreased signalling was seen after mutation of individual sites in CD4^+^ T cells. Thus, modulation of signalling strength by alternative MALT1 splicing seems to be more important in primary T cells, presumably to enhance weaker TCR signals. Constitutively expressed MALT1B seems to function as a molecular rheostat that limits the signalling strength in naive CD4^+^ T cells and MALT1A induction in activated T cells is required to overcome a threshold and to achieve a full response. Thus, we propose that TCR-induced MALT1 splicing is part of a positive feed-forward mechanism that is tuning T-cell signalling to allow productive T-cell activation.

MALT1 acts as a molecular scaffold protein to recruit the IKK complex to the CBM complex^5^, but it is also a protease which is activated on T-cell stimulation^6^,^7^. Since exon7 contains a functional TRAF6-binding site and TRAF6-dependent MALT1 ubiquitination drives NF-κB signalling^5^, the data suggest that exon7 inclusion augments MALT1 scaffolding. The E3 ligase TRAF6 can catalyse MALT1 poly-ubiquitination and trigger IKK activation^3^,^5^, but its role in T-cell activation has been questioned as TRAF6 ablation does not significantly affect TCR signalling to NF-κB and MAPK^29^. Nevertheless, our mutagenesis underscores an essential requirement of the TRAF6-binding motifs for MALT1 scaffolding function, suggesting that another E3 ligase may compensate for the loss of TRAF6. Clearly, inclusion of exon7 in MALT1A does not enhance protease function, which is in line with the results obtained by comparing *Malt1*^−/−^ and MALT1 paracaspase mutant (*Malt1*^*PM*^) mice^21^,^22^,^23^,^30^. CD4^+^ T cells from both *Malt1*^−/−^ and *Malt1*^*PM*^ mice are unable to differentiate into T_H_17 cells^22^,^24^. Congruently, we show in reconstitution experiments *in vitro* that catalytically inactive MALT1 does not promote T_H_17 development, but both MALT1 splice variants support T_H_17 differentiation. Further, our data indicate that even though T_H_1 differentiation is not completely relying on MALT1, the catalytic activity augments T_H_1 development. Thus, differentiation processes are apparently primarily driven by MALT1 protease function, which is required to remove the critical post-transcriptional regulators Roquin and Regnase-1 that counteract upregulation of T_H_17 differentiation factors such as IκBζ or IκBNS^12^,^31^,^32^,^33^. In contrast, proteolytic activity of MALT1 is largely dispensable for direct downstream signalling to NF-κB and JNK^21^,^22^,^30^, providing further evidence that the differential effects of MALT1 splice variants are caused by the scaffolding function. JNK activation has been connected to MALT1-catalysed CYLD cleavage^10^. However, MALT1 activity and CYLD cleavage were not affected by blocking MALT1A induction and JNK activation was also not impaired in T cells from *Malt1*^*PM*^ mice^21^,^22^,^30^, revealing that JNK signalling in naive CD4^+^ T cells is not relying on MALT1 protease activity. Thus, biochemical and functional evidence demonstrates that alternative splicing modulates MALT1 scaffolding function, but not protease activity.

We identified several RNA-binding factors that influence splicing of MALT1 by either promoting inclusion (for example, hnRNP R, hnRNP LL and SRSF3) or skipping (for example, hnRNP H1, hnRNP A1, U2AF1 and hnRNP U) of exon7. Together with a minigene approach, the results indicate that MALT1 exon7 splicing is controlled by a complex machinery of positive and negative *trans*-acting factors as well as *cis*-acting RNA elements. In the screen, we identified hnRNP U as the most potent negative regulator of exon7 inclusion in Jurkat T cells. hnRNP U knockdown enhanced TCR-induced upregulation of MALT1A and T-cell activation, supporting the conclusion that alternative splicing of MALT1 modulates T-cell responses. hnRNP U is able to bind DNA and RNA, and it has been implicated in a variety of biological processes. Recent genome-wide studies revealed that hnRNP U is an important regulator of alternative splicing^34^,^35^. hnRNP U can promote exon skipping or inclusion in a highly context-dependent manner and it can act in conjunction with other splicing regulators. Since the initial search already indicated that multiple RNA-binding proteins impact on alternative MALT1 splicing, it seems reasonable to assume that MALT1A and MALT1B levels will be regulated by a network of splice factors. TCR stimulation induces the expression of hnRNP U, but at the same time putative MALT1A promoting splice factors such as hnRNP R, SRSF3 and SRSF9 are increased, suggesting that expression and competition of a complex network of positive and negative splice factors may control MALT1 splicing. Clearly, a more detailed understanding about these factors and their *cis*-acting elements is required to elucidate the mechanism how TCR signalling impacts on MALT1 splicing. hnRNP U was suggested to regulate splice site selection either by modulating the core splicing machinery or by directly targeting the pre-mRNA^35^,^36^. The processes are not mutually exclusive and future studies must determine the exact mechanism how hnRNP U regulates exon7 splicing of MALT1. Little is known how cellular signalling pathways influence splicing. AKT signalling was shown to control hnRNP U- and hnRNP L-dependent Caspase-9 splicing^36^. Recently, JNK signalling was shown to induce alternative splicing of multiple targets including the MAP kinase kinase 7 (MKK7) upon T-cell activation^37^. It will be interesting to unravel if similar molecular mechanisms link TCR stimulation to MALT1 splicing.

We show that alternative MALT1 splicing impacts on signalling strength and induction of the hyperactive splice variant MALT1A pushes more robust T-cell activation. Such positive feed-forward regulation may facilitate to surpass thresholds for cellular decisions and they even can take place on the single-cell level. Thus, the relative MALT1A and MALT1B expression may trigger different programs in individual cells. Interestingly, intrinsic characteristics of T-cell signalling determine the fate of naive CD4^+^ T cells^38^. Strong TCR signalling favours T_FH_ over T_H_1 development, but the underlying molecular mechanisms that translates the magnitude of TCR signalling into a response remains elusive. We find that induced MALT1 splicing augments TCR signalling to NF-κB and JNK, and that MALT1A expression *in vivo* is indeed high in T_FH_ cells. We propose that MALT1 alternative splicing constitutes an early bifurcation point to translate TCR signalling into different T-cell activation states that may favour distinct fate decisions and effector functions in an immune response.

## Methods

### Mice

C57BL/6 mice were from Jackson laboratory and BALB/c as well as OT-II (C57BL/6Tg(TcraTcrb)425Cbn/Crl) mice were from Charles River. *Malt1*^−/−^ and *Malt1*^−/−^R26*/*CAG-CARΔ1^stop-fl^*Cd4*-Cre mice (C57BL/6 genetic background) were described^1^,^25^. All experiments were performed using 8–16-week-old (C57BL/6, BALB/c, OT-II) or 10–30-week-old (*Malt1*^−/−^ and *Malt1*^−/−^R26*/*CAG-CARΔ1^stop-fl^*Cd4*-Cre) male or female mice. All mice were housed in accordance with institutional guidelines. Immunization was done in accordance with established guidelines of the Regional Ethics Committee of Bavaria and animal protocols were approved by local authorities.

### Antibodies and reagents

The following antibodies were used for stimulation, fluorescence-activated cell sorting FACS) staining and western blot and IP: anti-CD3 (145-2C11), anti-CD28 (37.51), Streptavidin-PE, anti-CXCR5 (RF8B2, unconjugated) and anti-NEMO (Clone 54; western blot; all from BD Biosciences); anti-Thy1.1-APC (HIS51), anti-IL-2-FITC or anti-IL-2-APC (JES6-5H4), anti-CD25-PE (PC61.5), anti-CD69-APC (H1.2F3), anti-CD2-APC (RPA-2.10), anti-TCR-β-Biotin (H57-597), anti-CD4-PerCP (RM4-5) and anti-PD-1-FITC (J43; all from eBioscience); anti-BCL10 (EP606Y), biotinylated goat anti-rat IgG, anti-hnRNP U (3G6) and anti-TRAF6 (EP591Y; all from Abcam); anti-CD62L-AlexaFluor 647 (MEL-14; from Biolegend); anti-CD44-AlexaFluor 405 (IM7; Caltag); anti-MALT1 (B12), anti-MALT1 (H300), anti-β-Actin (I-19), anti-p38 (C-20), anti-CYLD (E10) and anti-NEMO (FL-419; IP; all from Santa Cruz Biotechnology); anti-CARMA1 (1D12), anti-MALT1 (2494), anti-p-ERK (9101), anti-p-p38 (9211), anti-p-JNK (9255), anti-p-IκBα (9246), anti-IκBα (L35A5), anti-JNK (9252) (all from Cell signalling); anti-ERK (442704; Calbiochem); rabbit anti-syrian hamster IgG (dianova) and anti-Flag-M2 (from Sigma-Aldrich); horseradish peroxidase (HRP)-conjugated secondary antibodies (Jackson ImmunoResearch); and rabbit polyclonal antibody was raised against peptide GRTDEAVEC from MALT1A. For differentiation experiments, the following antibodies, reagents and cytokines were used: anti-CD3 (145-2C11), anti-CD28 (37N), anti-IL-12 (C17.8), anti-IL-4 (11B11), anti-IFN-γ (Xmg1.2) and anti-CD16/32 (2.4G2; produced by V.H. and E. Kremmer); anti-mIL-2 (JES6-5H4; Miltenyi); anti-CD45.2-APC-Cy7 (30-F11), anti-CD4-PE-Cy7 (GK1.5), anti-IFN-γ-APC (XMG1.2), anti-IL-17A-PE (eBio17B7; all from eBioscience); indol-1 for live/dead-cell viability assays (Life Technologies); recombinant mouse IL-12 (BD Biosciences) and recombinant mouse IL-6 and TGF-β (R&D Systems). The following reagents were used: recombinant human TNF-α (Biomol); Phorbol 12-myristate 13-acetate (PMA/P) and ionomycin (Iono/I; both from Calbiochem); murine anti-CD3/CD28 coated beads (anti-CD3: 13.3 μg ml^−1^; anti-CD28: 26.6 μg ml^−1^; Life Technologies); human T-Activator CD3/CD28 Dynabeads (Life Technologies); Protein G Sepharose (GE Healthcare); Strep-Tactin Sepharose (IBA); brefeldin-A (Sigma); OVA peptide (aa 323–339; ISQAVHAAHAEINEAGR; Biotrend), LumiGlo reagent (Cell Signaling), vivo-MOs: AMO, GAACCAAAGGATTGCACTACCTTCA and standard control (cMO), CCTCTTACCTCAGTTACAATTTATA (both from GeneTools). For siRNA knockdown experiments, the following siRNAs and shRNAs were used: ON-TARGET*plus* SMARTpool siRNA library (qPCR) and siGENOME SMARTpool siRNA library (radioactive PCR; all from Dharmacon). ON-TARGET*plus* Non-targeting pool (si-control) and ON-TARGET*plus* SMARTpool si-hnRNP U were used for further knockdown experiments. For verification of the SMARTpool effects, the following individual siRNAs were used: siRNA negative control (si-control), si-hnRNP U #1: 5′-ACAGAAAGGCGGAGAUAAAUU-3′, si-hnRNP U #2: 5′-GAAGAAAGAUUGUGAAGUUUU-3′; si-hnRNP U #3: GAUGAAGACUAUAAGCAAAUU (all Eurogentec). For adenoviral knockdown experiments, shRNAs directed against hnRNP U (sh-hnRNP U #1: 5′-CCATAACTGTGCAGTTGAATT-3′, sh-hnRNP U #2: 5′-GGCTGGTCACTAACCACAAGT-3′) and adenoviral control virus were purchased from Sirion Biotech. The following oligonucleotides were used for electrophoretic mobility shift assays: H2K (forward: 5′-GATCCAGGGCTGGGGATTCCCCATCTCCACAGG-3′, reverse: 5′- GATCCCTGTGGAGATGGGGAATCCCCAGCCCTG-3′), OCT1 (forward: 5′- GATCTGTCGAATGCAAATCACTAGAA-3′, reverse: 5′-GATCTTCTAGTGATTTGCATTCGACA-3′).

### Cell culture and stimulation

Jurkat T cells were cultured in RPMI 1640 medium (Life Technologies) supplemented with 10% FCS and 100 U ml^−1^ penicillin/streptomycin (P/S, Life Technologies). Stimulation of Jurkat T cells was initiated by the addition of Phorbol 12-myristate 13-acetate (PMA) (200 ng ml^−1^) and ionomycin (300 ng ml^−1^) or TNF-α (10 ng ml^−1^). DMEM with 10% FCS, 100 U ml^−1^ P/S and 50 μM β-mercaptoethanol (Life Technologies) was used for culture of Phoenix packaging cells. Human embryonic kidney (HEK) 293 cells and MEFs were cultured in DMEM with 10% FCS and 100 U ml^−1^ P/S. For retroviral reconstitution experiments, CD4^+^ T cells from spleen and lymph nodes were isolated using mouse CD4^+^ T-cell-specific Dynabeads (Life Technologies). Differentiation experiments were performed using the naive CD4^+^ T-cell isolation kit (Miltenyi). For other experiments, isolation of murine CD4^+^ T cells was performed by negative magnetic-activated cell sorting (MACS) selection using the CD4^+^ T-cell isolation kit II (Miltenyi). Primary T cells were cultured in RPMI medium supplemented with 10% heat-inactivated FCS, 1% P/S, 1% NEAA (Life Technologies), 1% HEPES, 1% L-glutamine, 1% sodium pyruvate (Life Technologies) and 0.1% β-mercaptoethanol. CD4^+^ T cells were stimulated with anti-CD3 (0.5 μg ml^−1^) and anti-CD28 (1 μg ml^−1^) on plates precoated with rabbit anti-hamster IgG or anti-CD3/CD28-coated beads (3:1) or with PMA (30 ng ml^−1^) and ionomycin (300 ng ml^−1^). To analyse the isoform-specific effects regarding T_H_1 and T_H_17 differentiation, infected naive CD4^+^ T cells (0.2 × 10^6^) were stimulated with anti-CD3 (1 μg ml^−1^) and irradiated APCs (2 × 10^6^) from CD45.1 mouse (T:APC ratio is 1:10) in T_H_1 and T_H_17 culture conditions: T_H_1, IL-12 (10 ng ml^−1^) and anti-IL-4 (10 μg ml^−1^); T_H_17, TGF-β (10 μg ml^−1^), IL-6 (60 ng ml^−1^), anti-IL-12 (10 μg ml^−1^), anti-IL-4 (10 μg ml^−1^), anti-IFN-γ (5 μg ml^−1^) and anti-IL-2 (2.5 μg ml^−1^). Cells were cultured for 72 h until restimulation with PMA (20 nM) and ionomycin (1 μM) for 4 h. For restimulation, cells were washed twice with PBS and were incubated for 4 h with PMA (20 nM) and ionomycin (1 μM). To analyse differences in surface marker expression after adenoviral knockdown, cells (0.1–0.5 × 10^6^) were stimulated using anti-CD3 precoated 96-well plates (0.5 μg ml^−1^) and soluble anti-CD28 (1 μg ml^−1^). To determine MALT1 splicing in CD4^+^ T cells from OT-II mice, APCs were prepared from spleen and lymph node cells of wild-type mice and depleted for TCR-β^+^ T cells (using anti-TCR-β-Biotin). After irradiation (2,000 rad), APCs (2 × 10^6^) were pulsed with 1 μg ml^−1^ OVA peptide (aa 323–339) for 1 h at 37 °C, washed with PBS and subsequently incubated with OT-II CD4^+^ T cells (2 × 10^6^) in a ratio 1:1 for 6 h before being lysed in RNA lysis buffer. Human CD4^+^ T cells were isolated from peripheral blood by negative MACS selection (Miltenyi), cultured in advanced RPMI 1640 medium supplemented with 10% heat-inactivated FCS and 2 mM L-glutamine (Life Technologies), and stimulated (2 × 10^6^) with human T-Activator CD3/CD28 Dynabeads (Life Technologies). Written consent and approval by the ethics board of the Medical Faculty at the Technical University Munich was obtained for the use of peripheral blood from healthy donors.

### Generation and reconstitution of MALT1-deficient T cells

Bicistronic expression vector px330 expressing Cas9 and sgRNA^39^,^40^ was digested with *BbsI* and the linearized vector was gel purified. A pair of oligos (5′-CCGTGGTCCAGATATATAGC-3′ and 5′-GGTTGAAGCAAATGCAATGC-3′) for each targeting site was annealed and ligated to the linearized vector. Jurkat T cells (2.5 × 10^6^) were electroporated (220 V and 1,000 mA (Gene pulser X, Bio-Rad) with px330 plasmids expressing sgRNA targeting both the sides of the exon2 of the *MALT1* gene as well as plasmid bearing a puromycin selection cassette. At 24 h after electroporation, puromycin (2 μg ml^−1^) was added and taken off after 48 h. Isolation of clonal cell lines was achieved by serial dilutions and was followed by an appropriate expansion period. Cell clones were genotyped using PCR with intronic primers flanking both the sides of exon 2. MALT1 expression in exon2-deleted cells was analysed by western blot. For reconstitution, MALT1 isoforms were linked to Flag-Strep-Strep tag and hΔCD2 by a co-translational processing site T2A^41^ and introduced into pHAGE plasmids. Lentivirus was produced by transfecting sub-confluent HEK293T cells with 1.5 μg PAX, 1 μg pMD2G and 2 μg transfer vector. After 72 h, lentivirus was collected, filtered and Jurkat T cells (0.5 × 10^6^ cells) were infected in the presence of 8 μg ml^−1^ polybrene. After 24 h, virus was replaced with RPMI medium. After 1 week in culture, FACS analysis revealed >95% ΔCD2-positive cells.

### Retroviral and adenoviral transduction of CD4^+^ T cells

Retroviruses were produced in Phoenix cells transfected with pMSCV plasmids carrying human Flag-MALT1 constructs and Thy1.1 (separated by internal ribosome entry site (IRES) sequence) and viruses were collected after 48 and 72 h. For retroviral reconstitution, CD4^+^ T cells from *Malt1*^−al^ mice were simulated with plate-bound anti-CD3/anti-CD28 for 48 h and afterwards infected with retroviral supernatant supplemented with Polybrene (8 μg ml^−1^). After 6-h incubation, cells were resuspended in media supplemented with 20 U ml^−1^ IL-2 and expanded for another 72 h. Thy1.1-positive cells were purified by MACS (Miltenyi) yielding populations of ∼90% Thy1.1-positive T cells. Cells were then restimulated with plate-bound anti-CD3/anti-CD28 or P/I.

For T-cell differentiation experiments, full-length MALT1A, MALT1B or MALT1A C464A mutant were cloned in the pCAGAdDu-IRES-eGFP vector. HEK293A cells (1 × 10^5^) were transfected with 3 μg linearized plasmids using jetPEI reagent (Polyplus transfection). When all cells show cytopathic effects, cells were detached and primary virus was collected by three freeze–thaw cycles. Primary virus was amplified in HEK293A cells using an multiplicity of infection (MOI) of 50 and virus lysate was collected by freeze–thaw cycles after cytopathic effect. For adenoviral knockdown, adenoviruses encoding control or sh-hnRNP U #1 and #2 (identified above) were purchased from Sirion Biotech and amplified in HEK293A cells (200 μl). Virus was titrated using A549 cells. For differentiation experiments, naive CD4^+^ T cells from *Malt1*^−/−^R26*/*CAG-CARΔ1^stop-fl^*Cd4*-Cre mice were transduced with GFP control, MALT1A, MALT1B or MALT1A C464A virus using an MOI of 50. CD4^+^ T cells from R26*/*CAG-CARΔ1^stop-fl^*Cd4*-Cre mice were infected with control or sh-hnRNP U virus (MOI of 50). After 5 h of infection, cells were washed twice with PBS, resuspended in primary T-cell medium and rested for 40 h before sorting of GFP^+^ CD4^+^ T cells using FACS Star PLUS (BD) and stimulation. Transduction efficiency was determined by FACS. T-cell differentiation experiments were conducted as indicated above.

### Cell lysis and IP

For analysis of expression levels or activation of signalling pathways, cells (1–5 × 10^6^) were lysed in high-salt buffer (20 mM HEPES (pH 7.9), 350 mM NaCl, 20% glycerol, 1 mM MgCl_2_, 0.5 mM EDTA, 0.1 mM EGTA, 1% NP-40, 1 mM dithiothreitol (DTT), 10 mM sodium fluoride, 8 mM β-glycerophosphate, 300 μM sodium vanadate and protease inhibitor cocktail). For binding studies, cells (1–5 × 10^7^) were lysed in co-IP buffer (25 mM HEPES (pH 7.5), 150 mM NaCl, 0.2% NP-40, 10% glycerol, 1 mM DTT, 10 mM sodium fluoride, 8 mM β-glycerophosphate, 300 μM sodium vanadate and protease inhibitor cocktail). Lysate controls were mixed with 4 × SDS-loading dye and boiled. IP was carried out using antibodies against NEMO (9 μl) or MALT1A (15 μl) overnight at 4 °C. After antibody incubation, Protein G Sepharose (20 μl 1:1 suspension) was added and incubated for 1–2 h at 4 °C. For Strep-tagged proteins, precipitation was performed using Strep-Tactin Sepharose (30 μl 1:1 suspension) at 4 °C overnight. Beads were washed with co-IP buffer and boiled after addition of 20 μl 2 × SDS-loading dye. Lysates and IPs were separated by SDS–polyacrylamide gel electrophoresis (SDS–PAGE) and analysed by western blot.

### Western blot

Proteins were transferred onto polyvinylidene difluoride membranes for immunodetection using electrophoretic semi-dry transfer system. After transfer, membranes were blocked with 3% bovine serum albumin (BSA) for 1 h at room temperature and incubated with specific primary antibody (indicated above, dilution 1:1,000 in 1.5% BSA/PBS-T) overnight at 4 °C. Membranes were washed in PBS-T before addition of HRP-coupled secondary antibodies (indicated above, 1:7,000 in 0.75% BSA in PBS-T; 1 h, room temperature). HRP was detected by enhanced chemiluminescence using the LumiGlo reagent (Cell Signaling) according to the manufacturer's instructions. Images have been cropped for presentation. Full-size images are presented in Supplementary Fig. 7.

### Electrophoretic mobility shift assay

For electrophoretic mobility shift assays, double-stranded NF-κB or OCT1-binding sequences (H2K or OCT1 see reagents) were labelled with [α-^32^P] dATP using Klenow Fragment (NEB). To monitor DNA binding, whole-cell lysates (3–6 μg) were incubated for 30 min at room temperature with shift-buffer (HEPES (pH 7.9; 20 mM), KCl (120 mM), Ficoll (4%)), DTT (5 mM), BSA (10 μg) and poly-dI-dC (2 μg, Roche), and radioactive probe (10,000–20,000 c.p.m.). Samples were separated on a 5% polyacrylamide gel in TBE buffer, vacuum-dried and exposed to autoradiography. To determine NF-κB fold induction, intensities of NF-κB bands were quantified relative to OCT1 signal using ImageJ software. Images have been cropped for presentation. Full-size images are presented in Supplementary Fig. 7.

### Flow cytometry

For surface protein staining, unspecific binding was blocked with anti-CD16/32 (dilution 1:50) in FACS buffer (PBS with 2% FCS and 0.01% sodium azide) and cells were stained with monoclonal antibodies (dilution 1:100). For intracellular IL-2 staining, cells (0.5–1 × 10^6^) were stimulated for 6 h with anti-CD3/anti-CD28 in the presence of brefeldin-A (10 ng ml^−1^, Sigma) for 3 h before staining. Dead cells were excluded with ethidium monoazide bromide (dilution 1:100). Cells were fixed in 2% paraformaldehyde and permeabilized in PBS/0.5% Saponin/1% BSA. Unspecific binding was blocked with mouse anti-CD16/32 (dilution 1:50) and staining was performed with corresponding antibodies (indicated above, dilution 1:100). For T-cell differentiation experiments, cells were stimulated for 4 h with PMA (20 nM) and ionomycin (1 μM) in the presence of brefeldin-A for 2 h before staining. Afterwards, fixable dead-cell staining (indol-1) was performed for 30 min at 4 °C, followed by surface staining (anti-CD45.2, anti-CD4; dilution 1:200). Cells were fixed for 10 min at 20 °C with 4% paraformaldehyde and were permeabilized for 20 min at 4 °C in PBS/0.5% Saponin/1% BSA. The cells were then stained for 50 min at 4 °C with antibodies detecting IL-17A and IFNγ (dilution 1:200 each). Samples were acquired on LSRII or LSR Fortessa (BD) or Attune Acoustic Focusing Cytometer (Life Technologies) and analysed with FlowJo software (Tree Star).

### Immunization and isolation of different T-cell subsets

For isolation of different T-cell subsets, BALB/c mice were immunized intravenous with ∼2 × 10^8^ sheep erythrocytes (Acila AG, Mörfelden, Germany). After 1 week, the spleens were collected and single-cell suspensions prepared. To enrich for T cells, B cells were depleted using anti-CD19 microbeads (Miltenyi) according to the manual. Cells were then stained using following antibodies and reagents for 20 min on ice: anti-CXCR5, biotinylated goat anti-rat IgG, Streptavidin-PE, anti-CD4-PerCP, anti-PD-1-FITC, anti-CD62L-AlexaFluor 647 and anti-CD44-AlexaFluor 405. After washing, different T-cell subsets (CD4^+^CD62L^+^CD44^−^ naive T cells, CD4^+^CD62L^−^CD44^+^ effector T cells and CD4^+^CXCR5^+^PD1^+^ T_FH_ cells) were sorted on a FACS Aria cell sorter (BD) and subsequently lysed in Trizol for RNA extraction.

### MALT1 activity detection using MALT1-ABPs

MALT1-ABPs (6 and 7) have been described previously^19^. To investigate MALT1 activity of different MALT1-Strep constructs, Jurkat T cells (2–5 × 10^7^) were lysed in 900 μl co-IP buffer, centrifuged (14,000 r.p.m., 4 °C, 10 min) and overexpressed MALT1-Strep constructs were precipitated using Strep-Tactin Sepharose. After three washing steps with co-IP buffer, bead-bound MALT1-Strep was incubated with 3 μM fluorescent MALT1-ABP (probe 6: BODIPY-LRSR-AOMK) for 1 h at 30 °C, mixed with 4 × loading dye and boiled. Proteins were separated by SDS–PAGE and fluorescence was detected by a Typhoon Scanner (FITC laser 488 nm/filter 526 SP). A plate assay enzyme-linked activity sorbent assay was used to detect active MALT1 in primary CD4^+^ T cells. Cells (1 × 10^7^) were lysed in 200 μl co-IP buffer, centrifuged (14,000*g*, 4 °C, 10 min) and extract was incubated with 0.1 μM biotinylated MALT1-ABP (probe 7: BIOTIN-KLRSR-AOMK). Extract was transferred to streptavidin-coated plates (Thermo Scientific) and incubated overnight at 4 °C. After washing in PBS-T (0.05% Tween-20) and blocking with 2% BSA in PBS-T, detection was performed by incubation with MALT1 primary antibody (1:800 in 1% BSA in PBS-T; 1 h, room temperature) and HRP-coupled secondary antibody (1:1,500 in 1% BSA in PBS-T; 1 h, room temperature). Three to five washing steps with PBS-T were used after antibody incubation. Luminescence was determined after 3,3′,5,5′-Tetramethylbenzidin (TMB) substrate incubation for 10–20 min and subsequent stop reaction with 1 M H_2_SO_4_ in a photometer (Bio-Tek) at 450 and 570 nm.

### Cell transfection

Transfection of HEK293 cells with pEF Flag-MALT1 plasmids (2 μg) was performed by calcium phosphate transfection. For knockdown experiments using vivo morpholinos, CD4^+^ T cells (3 × 10^6^) or MEFs (0.5 × 10^6^) were transfected with 5 nmol of AMO or cMO and cultured for 3 h (RNA) or 18 h (protein). After MO transfection, primary T cells were stimulated with either plate-bound or bead-coupled anti-CD3/CD28 or P/I. Knockdown efficiency was determined by qPCR. For siRNA knockdown, Jurkat T cells were transfected with 100 nM siRNA and Atufect transfection reagent (0.5–1 μg ml^−1^; Silence Therapeutics) and analysed after 72 h. For minigene analysis, after 48 h of siRNA-transfecion, Jurkat T cells (2.5 × 10^6^) were electroporated with 1 μg minigene 1 (M1) or 2 (M2) using 220 V and 1,000 mA (Gene pulser X, Bio-Rad), cultivated for 24 h and subsequently lysed in protein or RNA lysis buffer. For siRNA screen using radioactive PCR, 1 × 10^6^ Jurkat T cells were transfected with siRNA (10 pmol, from siGenome SMARTpool) using the Amaxa Nucleofector and the manufacture's protocol for Jurkat T cells. RNA was analysed 60 h post transfection.

### RNA-binding protein IP and minigene

RIP was performed using the Magna RIP kit (Merck Millipore) according to the manual. Purified RNA was analysed by qPCR. RIP RNA fractions were normalized to the corresponding RNA input fractions and calculated relative to IgG control (IgG control set as 1). For the minigene assay, genomic *MALT1* region for M1 and M2 were amplified by PCR from human genomic clone pBACe3.6 (Source BioScience) and cloned between constant *CD45* exons (exon3 and exon7) in a minigene vector^42^. After transfection (as indicated above), RNA was isolated and reverse transcribed. To analyse MALT1 exon7 inclusion or exclusion of M1 and M2, two specific vector backbone primers (*CD45* exon3 forward and *CD45* exon7 reverse) were used to amplify alternatively spliced minigene products. RP2 levels served as internal control. PCR primers are listed in Supplementary Table 2 and 3.

### RNA preparation and qPCR

RNA was isolated (Qiagen RNeasy Kit) with subsequent DNase treatment (Promega) and reverse transcribed (Verso cDNA synthesis kit, Thermo Fisher). For qPCR, a LightCycler 480 from Roche was used with LightCycler SYBR Green I Master Mix. Semi-qPCR was performed using DreamTaq Polymerase (Thermo Fisher). For siRNA screen using MALT1 splicing-sensitive radioactive PCR, RNA was analysed with primers in flanking constant exons (ex6 and ex9/10). Products were analysed by denaturing PAGE and Phosphoimager quantification. PCR primers are listed in Supplementary Table 2 and 3.

## Additional information

**How to cite this article:** Meininger, I. *et al*. Alternative splicing of MALT1 controls signalling and activation of CD4^+^ T cells. *Nat. Commun.* 7:11292 doi: 10.1038/ncomms11292 (2016).

## Supplementary Material

Supplementary Information Supplementary Figures 1-7 and Supplementary Tables 1-3

## References

[b1] Ruland J., Duncan G. S., Wakeham A. & Mak T. W. Differential requirement for Malt1 in T and B cell antigen receptor signaling. Immunity 19, 749–758 (2003). 14614861 10.1016/s1074-7613(03)00293-0

[b2] Ruefli-Brasse A. A., French D. M. & Dixit V. M. Regulation of NF-kappaB-dependent lymphocyte activation and development by paracaspase. Science 302, 1581–1584 (2003). 14576442 10.1126/science.1090769

[b3] Sun L., Deng L., Ea C. K., Xia Z. P. & Chen Z. J. The TRAF6 ubiquitin ligase and TAK1 kinase mediate IKK activation by BCL10 and MALT1 in T lymphocytes. Mol. Cell 14, 289–301 (2004). 15125833 10.1016/s1097-2765(04)00236-9

[b4] Noels H. . A Novel TRAF6 binding site in MALT1 defines distinct mechanisms of NF-kappaB activation by API2middle dotMALT1 fusions. J. Biol. Chem. 282, 10180–10189 (2007). 17287209 10.1074/jbc.M611038200

[b5] Oeckinghaus A. . Malt1 ubiquitination triggers NF-kappaB signaling upon T-cell activation. EMBO J. 26, 4634–4645 (2007). 17948050 10.1038/sj.emboj.7601897 PMC2080808

[b6] Coornaert B. . T cell antigen receptor stimulation induces MALT1 paracaspase-mediated cleavage of the NF-kappaB inhibitor A20. Nat. Immunol. 9, 263–271 (2008). 18223652 10.1038/ni1561

[b7] Rebeaud F. . The proteolytic activity of the paracaspase MALT1 is key in T cell activation. Nat. Immunol. 9, 272–281 (2008). 18264101 10.1038/ni1568

[b8] Duwel M. . A20 negatively regulates T cell receptor signaling to NF-kappaB by cleaving Malt1 ubiquitin chains. J. Immunol. 182, 7718–7728 (2009). 19494296 10.4049/jimmunol.0803313

[b9] Hailfinger S. . Malt1-dependent RelB cleavage promotes canonical NF-kappaB activation in lymphocytes and lymphoma cell lines. Proc. Natl Acad. Sci. USA 108, 14596–14601 (2011). 21873235 10.1073/pnas.1105020108 PMC3167514

[b10] Staal J. . T-cell receptor-induced JNK activation requires proteolytic inactivation of CYLD by MALT1. EMBO J. 30, 1742–1752 (2011). 21448133 10.1038/emboj.2011.85 PMC3101995

[b11] Uehata T. . Malt1-induced cleavage of regnase-1 in CD4(+) helper T cells regulates immune activation. Cell 153, 1036–1049 (2013). 23706741 10.1016/j.cell.2013.04.034

[b12] Jeltsch K. M. . Cleavage of roquin and regnase-1 by the paracaspase MALT1 releases their cooperatively repressed targets to promote T17 differentiation. Nat. Immunol. 15, 1079–1089 (2014). 25282160 10.1038/ni.3008

[b13] Klein T. . The paracaspase MALT1 cleaves HOIL1 reducing linear ubiquitination by LUBAC to dampen lymphocyte NF-kappaB signalling. Nat. Commun. 6, 8777 (2015). 26525107 10.1038/ncomms9777 PMC4659944

[b14] Martinez N. M. & Lynch K. W. Control of alternative splicing in immune responses: many regulators, many predictions, much still to learn. Immunol. Rev. 253, 216–236 (2013). 23550649 10.1111/imr.12047 PMC3621013

[b15] Hong C. . Activated T cells secrete an alternatively spliced form of common gamma-chain that inhibits cytokine signaling and exacerbates inflammation. Immunity 40, 910–923 (2014). 24909888 10.1016/j.immuni.2014.04.020 PMC4143255

[b16] Magistrelli G. . A soluble form of CTLA-4 generated by alternative splicing is expressed by nonstimulated human T cells. Eur. J. Immunol. 29, 3596–3602 (1999). 10556814 10.1002/(SICI)1521-4141(199911)29:11<3596::AID-IMMU3596>3.0.CO;2-Y

[b17] Hermiston M. L., Xu Z. & Weiss A. CD45: a critical regulator of signaling thresholds in immune cells. Annu. Rev. Immunol. 21, 107–137 (2003). 12414720 10.1146/annurev.immunol.21.120601.140946

[b18] Rothrock C., Cannon B., Hahm B. & Lynch K. W. A conserved signal-responsive sequence mediates activation-induced alternative splicing of CD45. Mol. Cell 12, 1317–1324 (2003). 14636588 10.1016/s1097-2765(03)00434-9

[b19] Eitelhuber A. C. . Activity-based probes for detection of active MALT1 paracaspase in immune cells and lymphomas. Chem. Biol. 22, 129–138 (2015). 25556945 10.1016/j.chembiol.2014.10.021

[b20] Pelzer C. . The protease activity of the paracaspase MALT1 is controlled by monoubiquitination. Nat. Immunol. 14, 337–345 (2013). 23416615 10.1038/ni.2540

[b21] Jaworski M. . Malt1 protease inactivation efficiently dampens immune responses but causes spontaneous autoimmunity. EMBO J. 33, 2765–2781 (2014). 25319413 10.15252/embj.201488987 PMC4282555

[b22] Bornancin F. . Deficiency of MALT1 paracaspase activity results in unbalanced regulatory and effector T and B cell responses leading to multiorgan inflammation. J. Immunol. 194, 3723–3734 (2015). 25762782 10.4049/jimmunol.1402254

[b23] Yu J. W. . MALT1 Protease Activity Is Required for Innate and Adaptive Immune Responses. PLoS ONE 10, e0127083 (2015). 25965667 10.1371/journal.pone.0127083 PMC4428694

[b24] Brustle A. . The NF-kappaB regulator MALT1 determines the encephalitogenic potential of Th17 cells. J. Clin. Invest. 122, 4698–4709 (2012). 23114599 10.1172/JCI63528 PMC3590210

[b25] Heger K. . A novel Cre recombinase reporter mouse strain facilitates selective and efficient infection of primary immune cells with adenoviral vectors. Eur. J. Immunol. 45, 1614–1620 (2015). 25787118 10.1002/eji.201545457

[b26] Risso A. . CD69 in resting and activated T lymphocytes. Its association with a GTP binding protein and biochemical requirements for its expression. J. Immunol. 146, 4105–4114 (1991). 1710239

[b27] Thome M., Charton J. E., Pelzer C. & Hailfinger S. Antigen receptor signaling to NF-kappaB via CARMA1, BCL10, and MALT1. Cold Spring Harb. Perspect. Biol. 2, a003004 (2010). 20685844 10.1101/cshperspect.a003004 PMC2926749

[b28] Ip J. Y. . Global analysis of alternative splicing during T-cell activation. RNA 13, 563–572 (2007). 17307815 10.1261/rna.457207 PMC1831861

[b29] King C. G. . TRAF6 is a T cell-intrinsic negative regulator required for the maintenance of immune homeostasis. Nat. Med. 12, 1088–1092 (2006). 16921377 10.1038/nm1449

[b30] Gewies A. . Uncoupling Malt1 threshold function from paracaspase activity results in destructive autoimmune inflammation. Cell Rep. 9, 1292–1305 (2014). 25456129 10.1016/j.celrep.2014.10.044

[b31] Okamoto K. . I kappa B zeta regulates T(H)17 development by cooperating with ROR nuclear receptors. Nature 464, 1381–1385 (2010). 20383124 10.1038/nature08922

[b32] Kobayashi S. . The nuclear I kappa B family protein I kappa B-NS influences the susceptibility to experimental autoimmune encephalomyelitis in a murine model. PLoS ONE 9, e110838 (2014). 25347393 10.1371/journal.pone.0110838 PMC4210207

[b33] Annemann M. . I kappa B-NS Regulates Murine Th17 Differentiation during Gut Inflammation and Infection. J. Immunol. 194, 2888–2898 (2015). 25694610 10.4049/jimmunol.1401964

[b34] Huelga S. C. . Integrative genome-wide analysis reveals cooperative regulation of alternative splicing by hnRNP proteins. Cell Rep. 1, 167–178 (2012). 22574288 10.1016/j.celrep.2012.02.001 PMC3345519

[b35] Xiao R. . Nuclear matrix factor hnRNP U/SAF-A exerts a global control of alternative splicing by regulating U2 snRNP maturation. Mol. Cell 45, 656–668 (2012). 22325991 10.1016/j.molcel.2012.01.009 PMC3299905

[b36] Vu N. T. . hnRNP U Enhances Caspase-9 Splicing and Is Modulated by AKT-dependent Phosphorylation of hnRNP L. J. Biol. Chem. 288, 8575–8584 (2013). 23396972 10.1074/jbc.M112.443333 PMC3605676

[b37] Martinez N. M. . Widespread JNK-dependent alternative splicing induces a positive feedback loop through CELF2-mediated regulation of MKK7 during T-cell activation. Genes Dev. 29, 2054–2066 (2015). 26443849 10.1101/gad.267245.115 PMC4604346

[b38] Tubo N. J. . Single naive CD4^+^ T cells from a diverse repertoire produce different effector cell types during infection. Cell 153, 785–796 (2013). 23663778 10.1016/j.cell.2013.04.007 PMC3766899

[b39] Cong L. . Multiplex genome engineering using CRISPR/Cas systems. Science 339, 819–823 (2013). 23287718 10.1126/science.1231143 PMC3795411

[b40] Ran F. A. . Genome engineering using the CRISPR-Cas9 system. Nat. Protoc. 8, 2281–2308 (2013). 24157548 10.1038/nprot.2013.143 PMC3969860

[b41] Hadian K. . NF-kappaB essential modulator (NEMO) interaction with linear and lys-63 ubiquitin chains contributes to NF-kappaB activation. J. Biol. Chem. 286, 26107–26117 (2011). 21622571 10.1074/jbc.M111.233163 PMC3138271

[b42] Motta-Mena L. B., Heyd F. & Lynch K. W. Context-dependent regulatory mechanism of the splicing factor hnRNP L. Mol. Cell 37, 223–234 (2010). 20122404 10.1016/j.molcel.2009.12.027 PMC2818868

