# Supplementary material for: Alternative splicing of MALT1 controls signalling and activation of CD4+ T cells
Source: Nat Commun. 2016 Apr 12;7:11292. doi: 10.1038/ncomms11292 (PMC4832065; doi:10.1038/ncomms11292)
Supplement: Supplementary Information — Supplementary Figures 1-7 and Supplementary Tables 1-3 [file ncomms11292-s1.pdf]

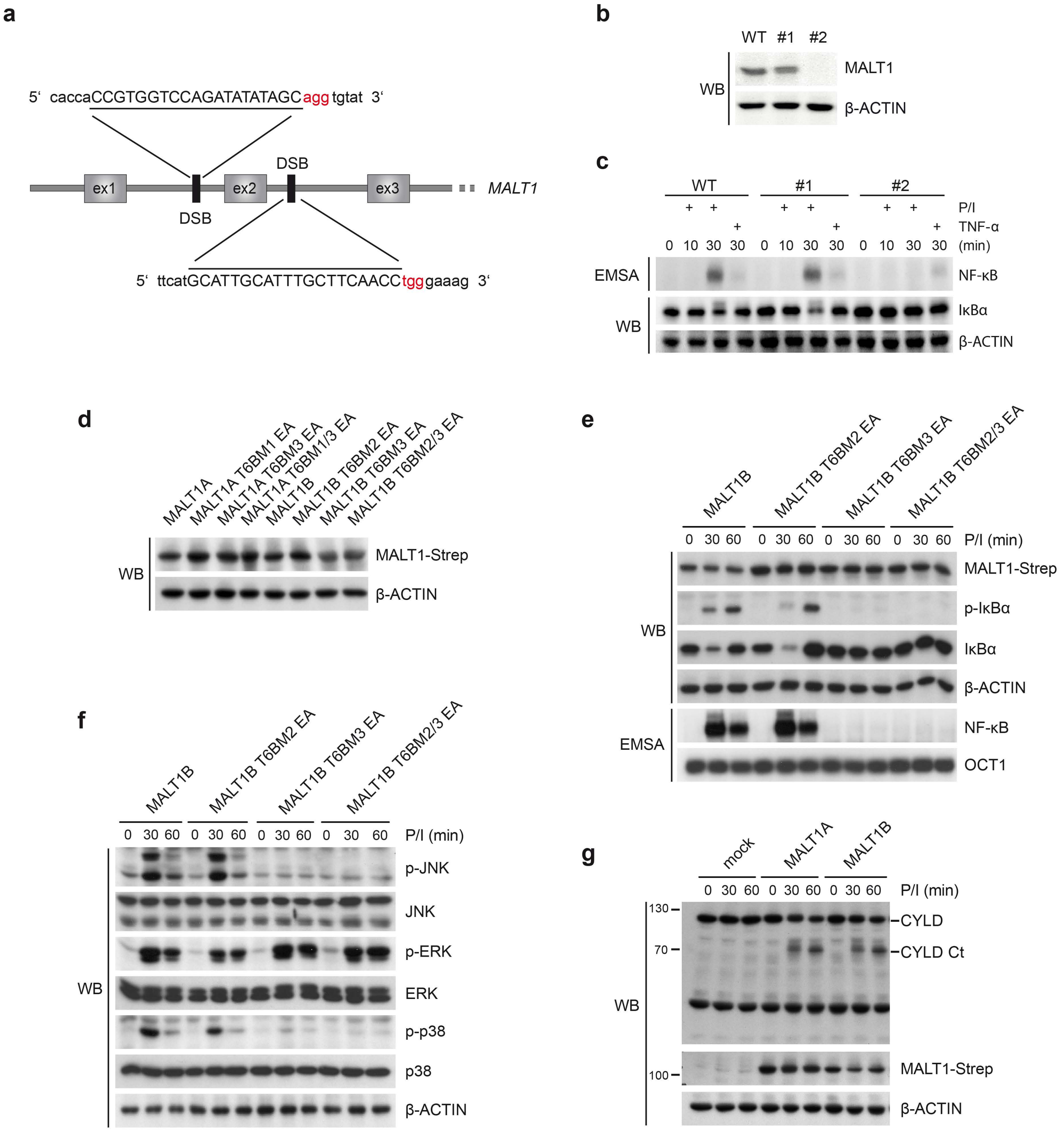

Supplementary Figure 1:

Generation of MALT1 knock-out in Jurkat T cells. (**a**) Schematic of the Cas9/sgRNA-targeting sites in the *MALT1* gene. The sgRNA-targeting sequences are underlined and the protospacer-adjacent motif (PAM) is labeled in red. Induced double-strand breaks are marked with dotted lines. (**b**) MALT1 expression in Jurkat T cell clones was analyzed by Western Blot. (**c**) WT, unaffected clone #1 and heterozygous clone #2 were stimulated with P/I or TNF-α for the indicated time points. NF-κB signaling was analyzed by EMSA and Western Blot. (**d-g**) MALT1-deficient Jurkat T cell clone was reconstituted with StrepTagII (mock) or MALT1-StrepTagII variants. (**d**) MALT1 expression was monitored by Western Blot. (**e,f**) Cells reconstituted with MALT1B wildtype or MALT1B mutants were stimulated for the indicated time points and analyzed for NF-κB and MAPK signaling by EMSA and Western Blot. (**g**) CYLD cleavage was monitored by Western Blot after P/I stimulation. Data are representative for three (**e-g**) independent experiments.

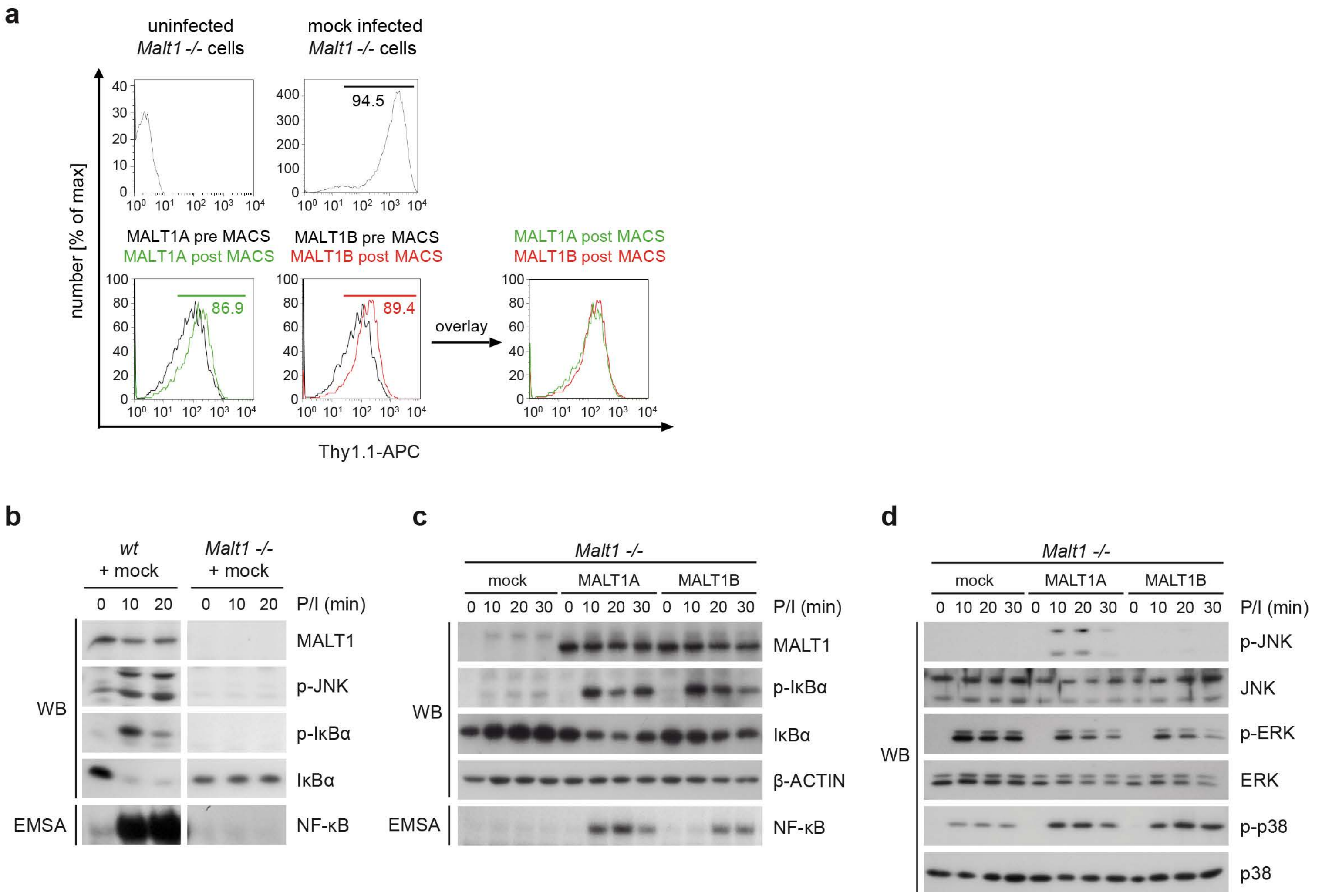

Supplementary Figure 2:

Retroviral transduction and stimulation of primary murine CD4$^+$ T cells. (**a**) FACS histograms showing transduction and the MACS enrichment of Thy1.1 positive reconstituted T cells. T cells of *Malt1*$^{-/-}$ mice were retrovirally reconstituted with MALT1 expression constructs and infection marker Thy1.1 on the surface. By magnetic cell sorting (MACS) of Thy1.1 positive cells, cell populations were 80-90% positive for Thy1.1. (**b**) CD4$^+$ T cells from wild type mice or *MALT1*$^{-/-}$ mice reconstituted with mock retroviruses were stimulated with P/I for the indicated time points. NF-κB and JNK signaling was monitored by Western Blot and EMSA. (**c**,**d**) CD4$^+$ T cells from *MALT1*$^{-/-}$ mice were retrovirally reconstituted either with mock, MALT1A or MALT1B. MALT1 expression, NF-κB activation (**c**) and MAPK signaling (**d**) were monitored by Western Blot after P/I treatment. Data are representative for three independent experiments.

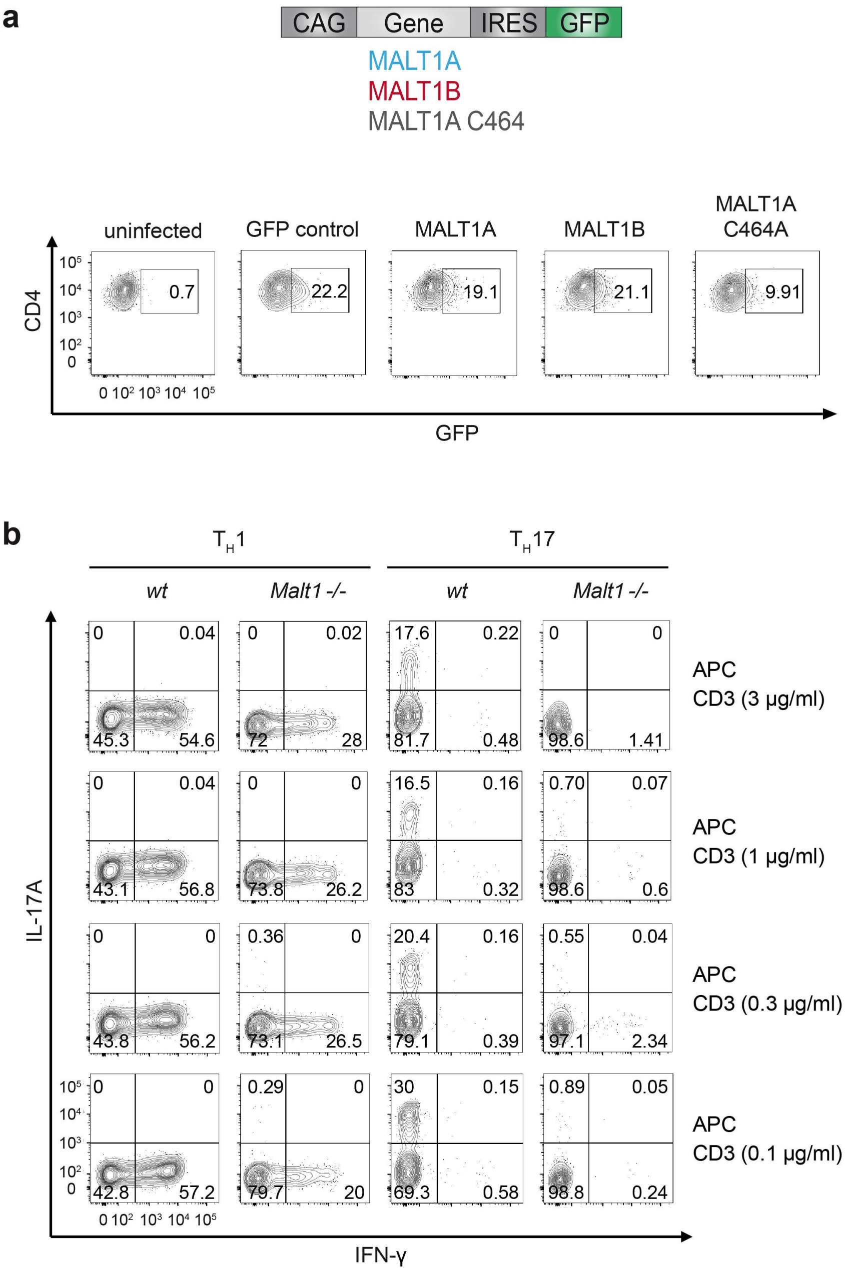

Supplementary Figure 3:

Adenoviral transduction and T_H1/T_H17 differentiation of MALT1 deficient T cells (**a**) Scheme of adenoviral CAG promotor constructs for MALT1 expression. CD4$^+$ T cells from *Malt1*$^{-/-}$ R26/CAG-CARΔ1$^{stop\text{-}fl}$*Cd4*-Cre mice were transduced with mock, MALT1A, MALT1B or MALT1A C464A expressing adenoviruses co-expressing IRES–GFP. Transduction was monitored by gating of CD4+ T cells on GFP positive T cells for further analysis. (**b**) CD4$^+$ T cells from *wt* and *Malt1*$^{-/-}$ mice were treated with APCs (ratio 1:10) and increasing concentrations of anti-CD3 antibodies under T_H1 or T_H17 skewing conditions. Intracellular expression of T_H1 marker IFN-γ and T_H17 marker IL-17A were determined by FACS. Data are representative of two (**b**) or four (**a**) independent experiments.

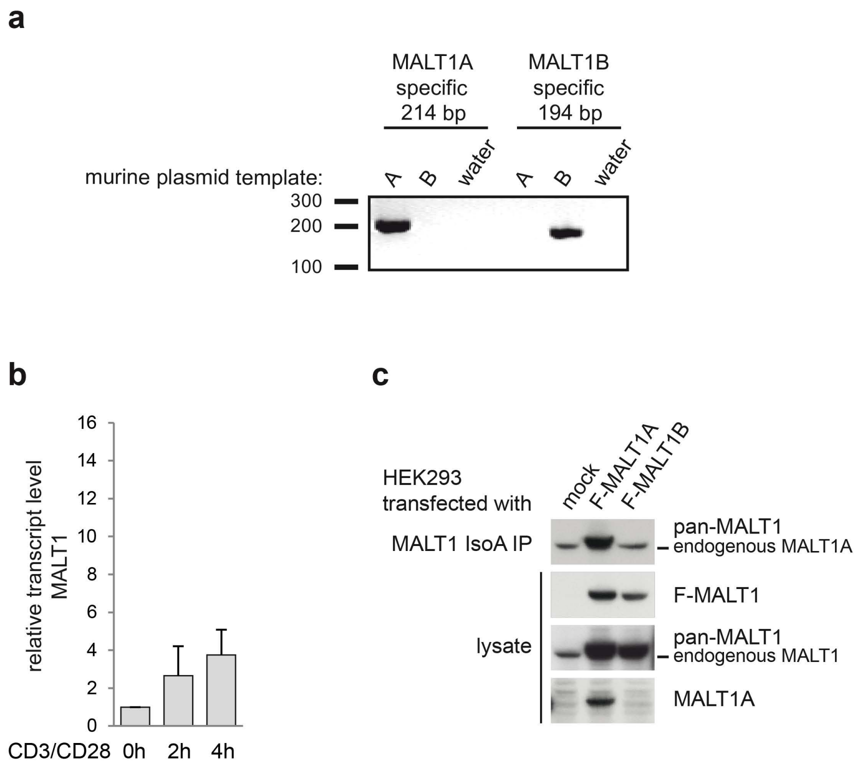

Supplementary Figure 4:

MALT1A/B primer and antibody specificity. (**a**) Specificity for MALT1A or MALT1B amplification using specific MALT1A (ex5-ex7/8) or MALT1B (ex5-ex6/8) primers was verified using murine MALT1 cDNA cloned into plasmid vector. (**b**) Total MALT1 mRNA levels of $CD4^+$ T cells from BALB/c mice were analyzed by qPCR using primer pair ex16-ex17 after anti-CD3/CD28 stimulation. HMBS was used for normalization. (**c**) HEK 293 cells were transfected with mock, Flag-MALT1A or Flag-MALT1B expression vectors. MALT1A specific antibody recognizes exclusively overexpressed MALT1A in the cell lysate. IP using MALT1A antibody was used to enrich Flag-MALT1A or endogenous MALT1A. Pan-MALT1 antibody (2494) was used for detection in Western Blot. Data are representative for two (**a, c**) or three (**b**) independent experiments.

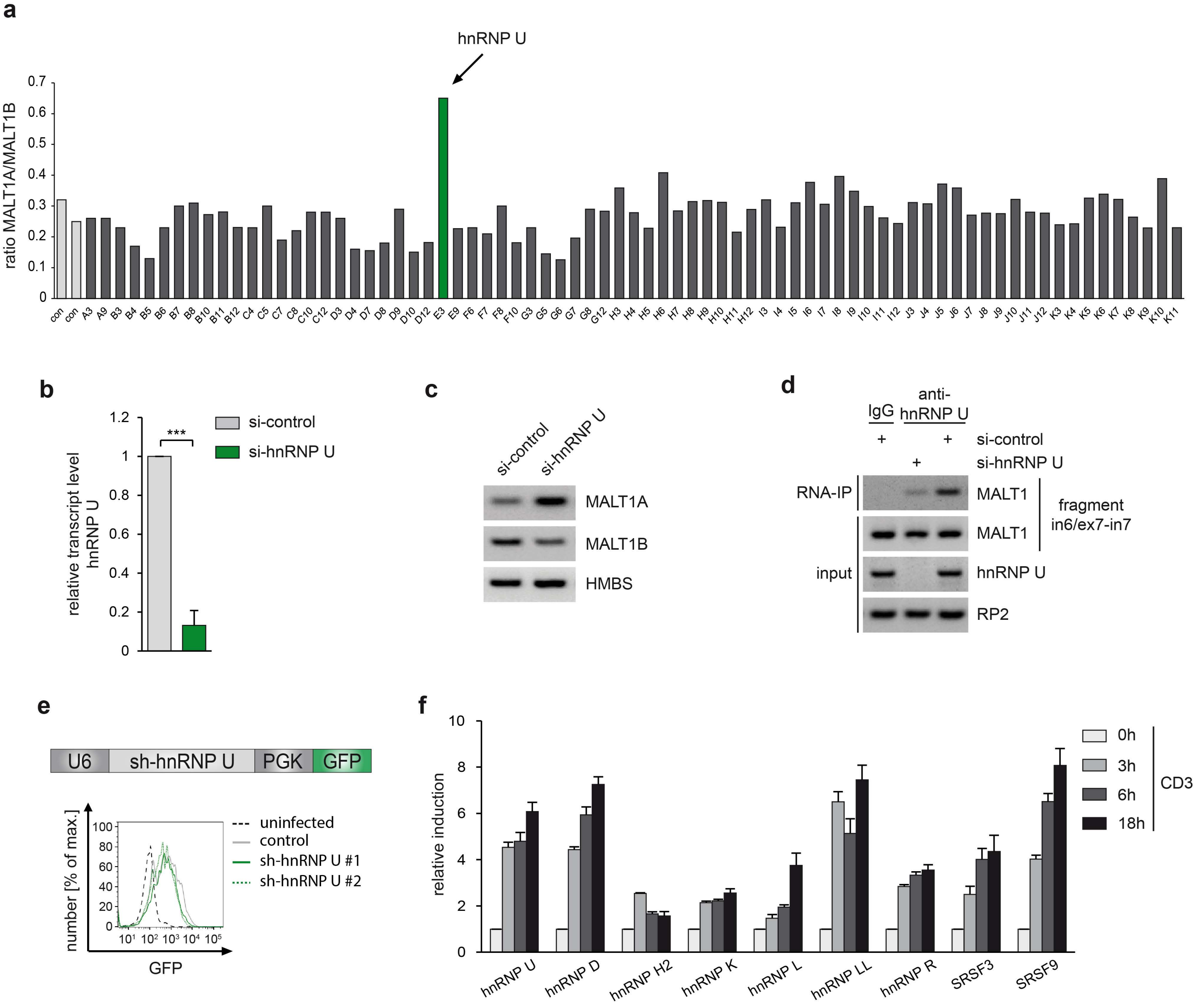

Supplementary Figure 5:

hnRNP U knock-down and adenoviral transduction of CD4^{+} T cells. (**a**) RNAi screen to identify regulators of alternative MALT1 splicing in Jurkat T cells. Cells were transfected with smart pool siRNA against putative splicing regulators and the ratio MALT1A/B mRNA was analyzed by radioactive PCR. (**b**) hnRNP U knock-down on mRNA level after transfection of smartpool siRNA (used in Fig. 5a) in Jurkat T cells. hnRNP U expression level was analyzed by qPCR in relation to si-control. (**c**) Effects of hnRNP U knock-down in Jurkat T cells on MALT1A and MALT1B expression analyzed by semi-qPCR. (**d**) Binding of hnRNP U to MALT1 pre-mRNA analyzed by semi-qPCR. IgG control IP or anti-hnRNP U IP was carried out from extracts of si-control or si-hnRNP U transfected Jurkat T cells. IP of MALT1 pre-mRNA was detected by semi-qPCR using primers amplifying the in6/ex7-in7 fragment. mRNAs for MALT1, hnRNP U and RP2 in the input were used as controls. (**e**) Scheme of adenoviral U6 promotor sh-hnRNP U knock-down construct (left). FACS showing GFP expression of CD4^{+} T cells from R26/CAG-CARΔ1$^{stop\text{-}fl}$*Cd4*-Cre mice transduced with control or sh-hnRNP U adenoviruses co-expressing GFP (right). (**f**) Analysis of mRNA levels of several hnRNP and SR proteins in CD4^{+} T cells from Balb/c mice after anti-CD3 stimulation by qPCR. HMBS served as internal control and relative induction was determined comparative to unstimulated cells. Data are representative for one (**a**), two (**d**,**e**) or three (**b**,**c**,**f**) independent experiments. (**b**) Depicted is the mean ± SD (n=3). ***$p < 0.001$; unpaired *t*-test.

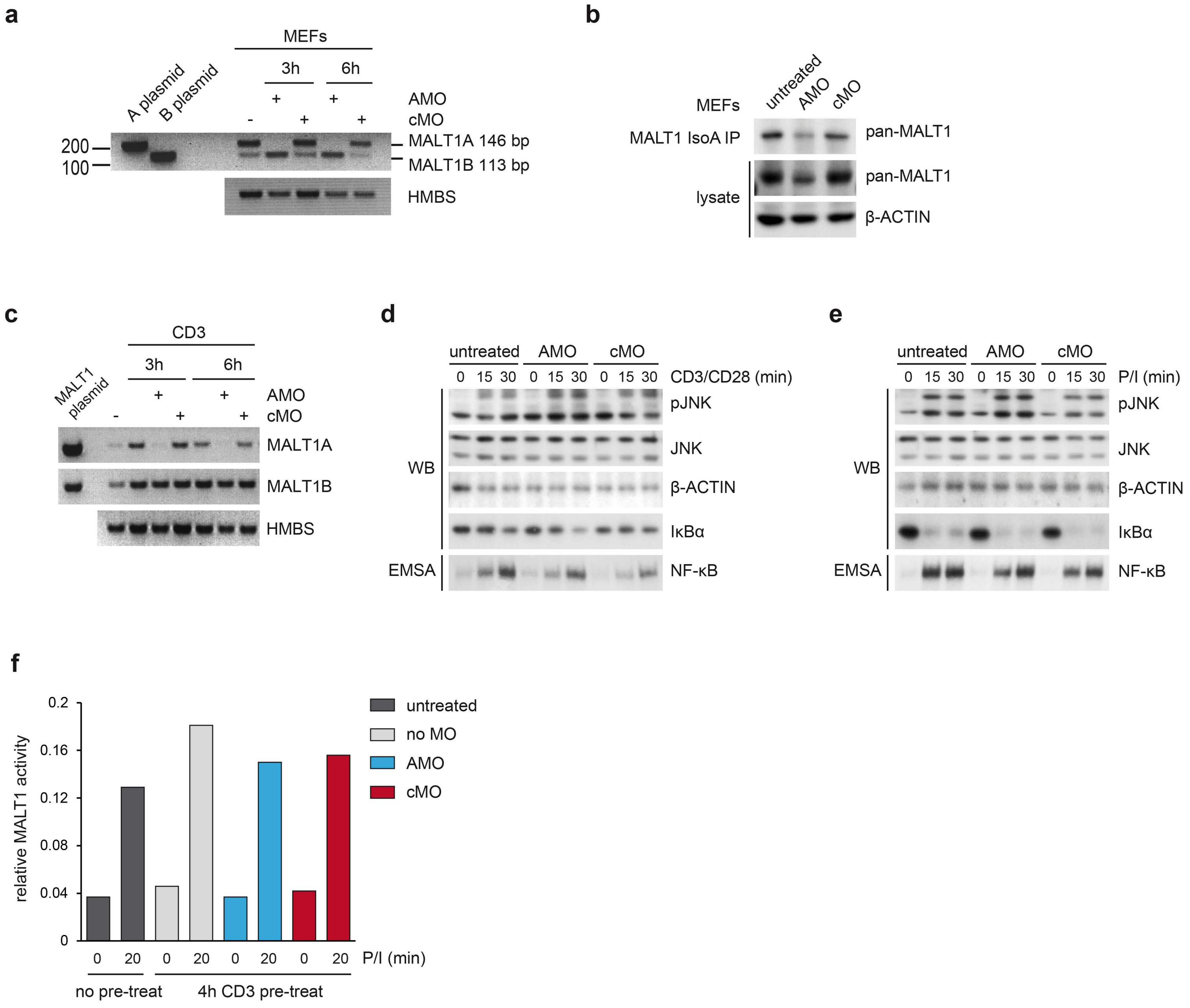

Supplementary Figure 6:

Morpholino knock-down of MALT1A. (**a**) MEFs were treated with MALT1A morpholino (AMO) or control morpholino (cMO) or left untreated for the indicated times. MALT1A and MALT1B expression was analyzed by semi-qPCR using primer pair ex6-ex9/10. HMBS and MALT1A and MALT1B plasmid DNA was used as control. (**b**) IP MALT1A in MEFs after treatment with AMO or cMO for 18 h. Western Blots were stained with pan-MALT1 antibody. (**c**) $CD4^+$ T cells from BALB/c mice were incubated with AMO or cMO for 3 h. Cells were stimulated and MALT1 isoform levels were analyzed by semi-qPCR using primer pairs ex5-ex7/8 or ex5-ex6/8. (**d, e**) Untreated and MO-treated cells were stimulated with anti-CD3/CD28 or P/I and were monitored for JNK phosphorylation and IκBα degradation by Western Blot and NF-κB activation by EMSA. (**f**) $CD4^+$ T cells from BALB/c mice were incubated with AMO or cMO for 18 h. After CD3 pre-treatment and re-stimulation with P/I, active MALT1 was detected using a biotinylated MALT1-ABP probe. MALT1 activity was calculated relative to background levels. Data are representative for two (**d**,**e**) or three (**a**-**c**) independent experiments.

Figure 1c

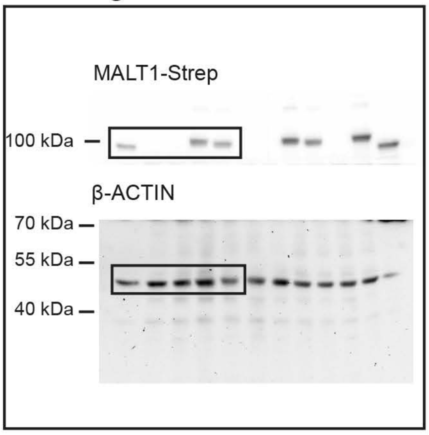

Figure 1d

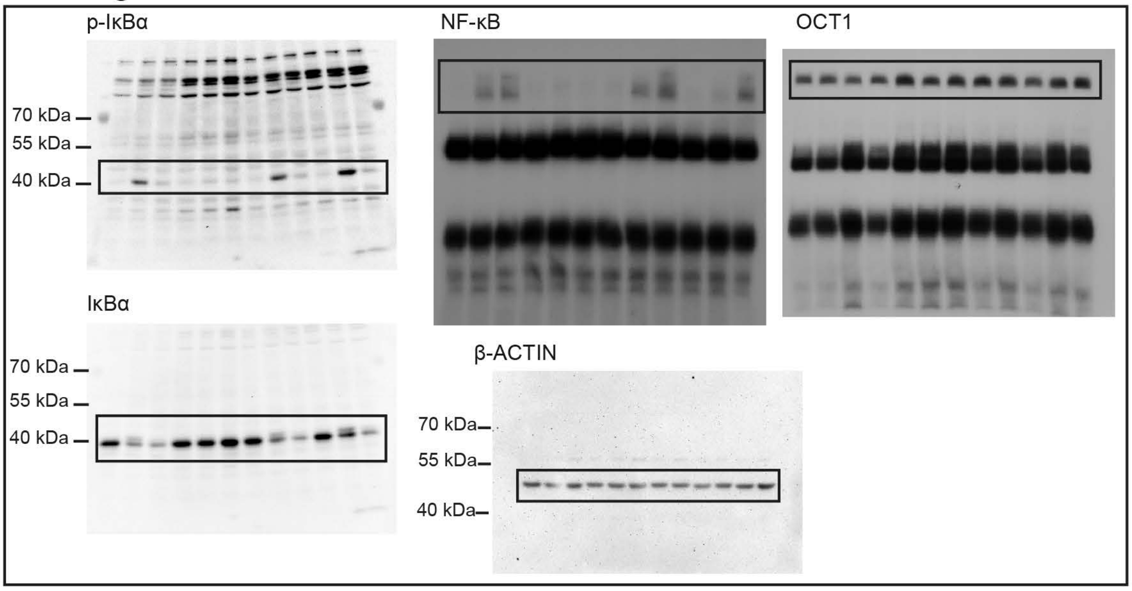

Figure 1e

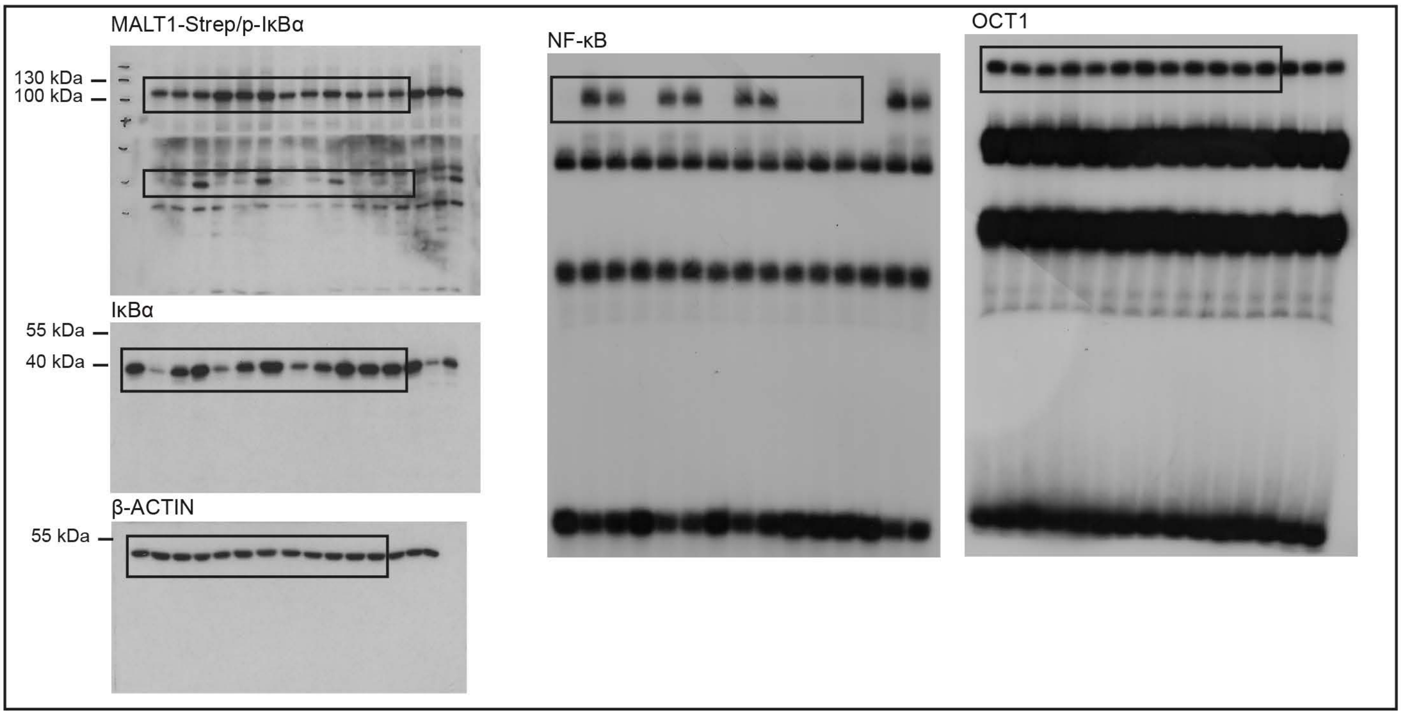

Figure 1f

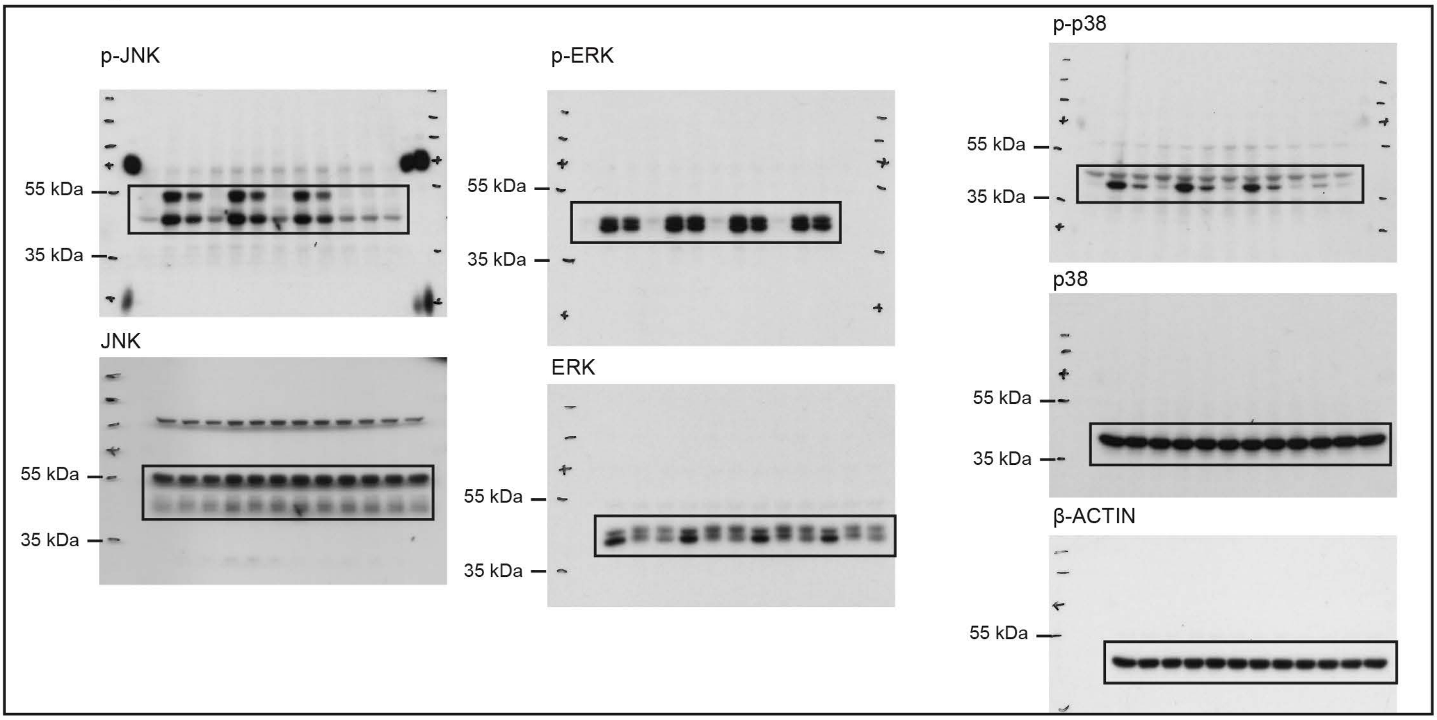

Supplementary Figure 7:

Uncropped images. Black boxes show approximate image size used for presentation.

Figure 1g

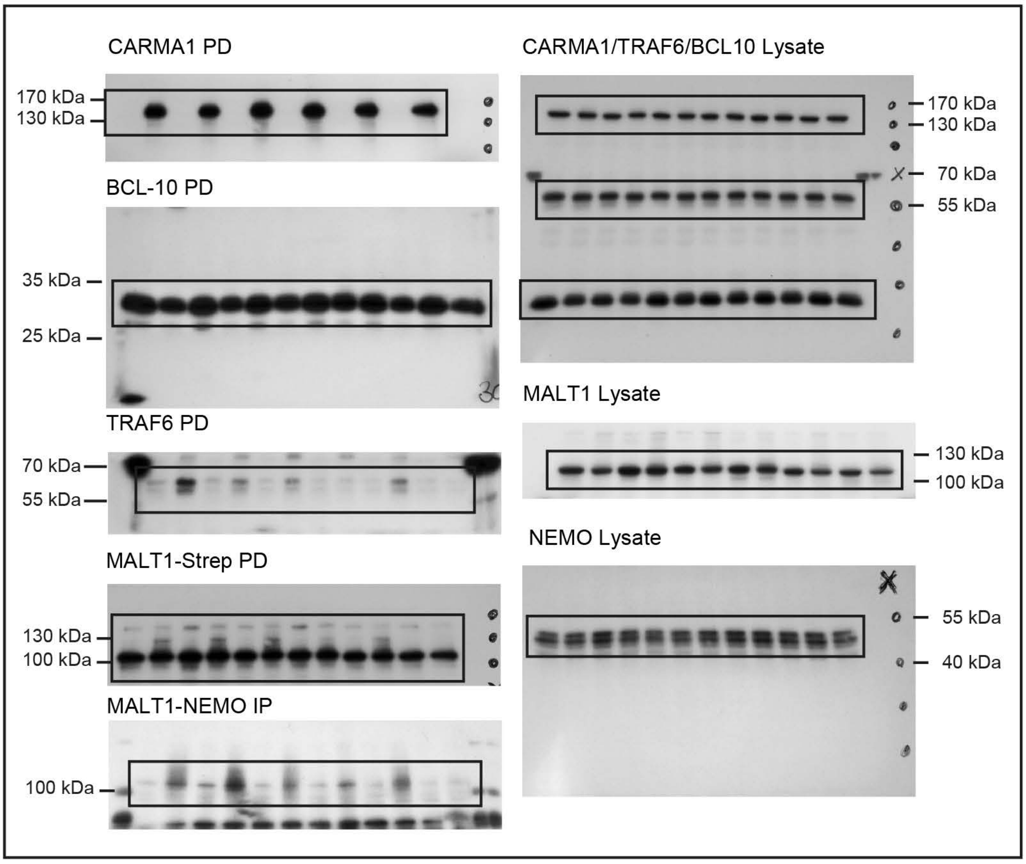

Figure 1h

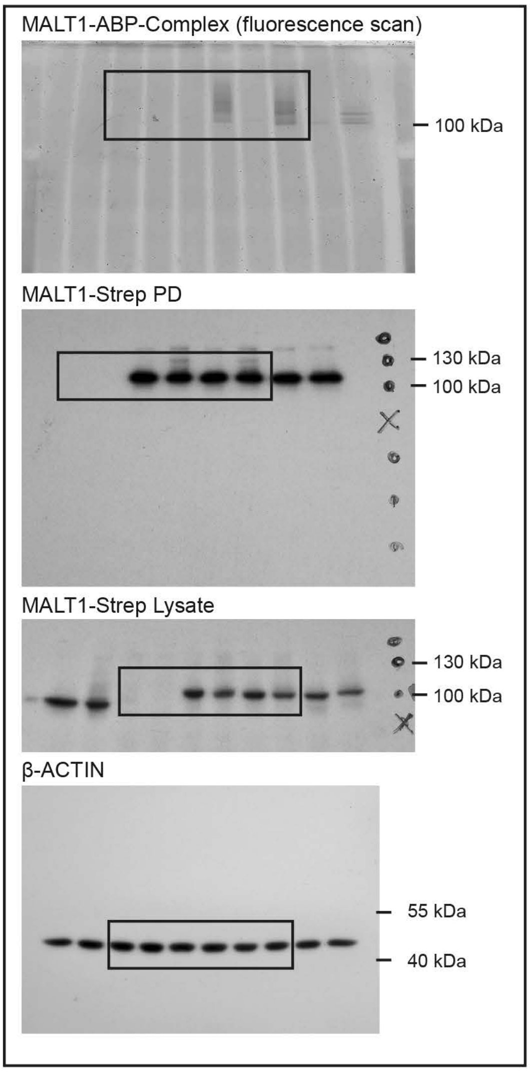

Figure 2a

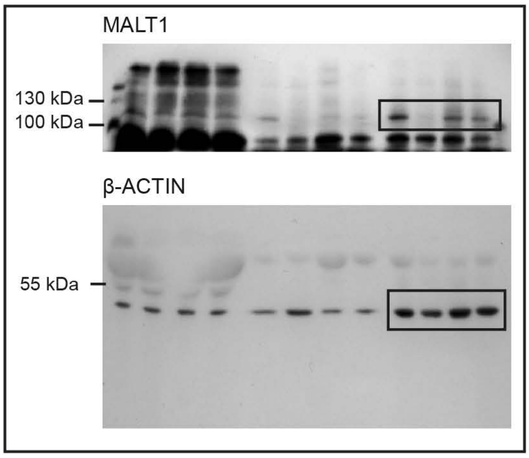

Figure 2b

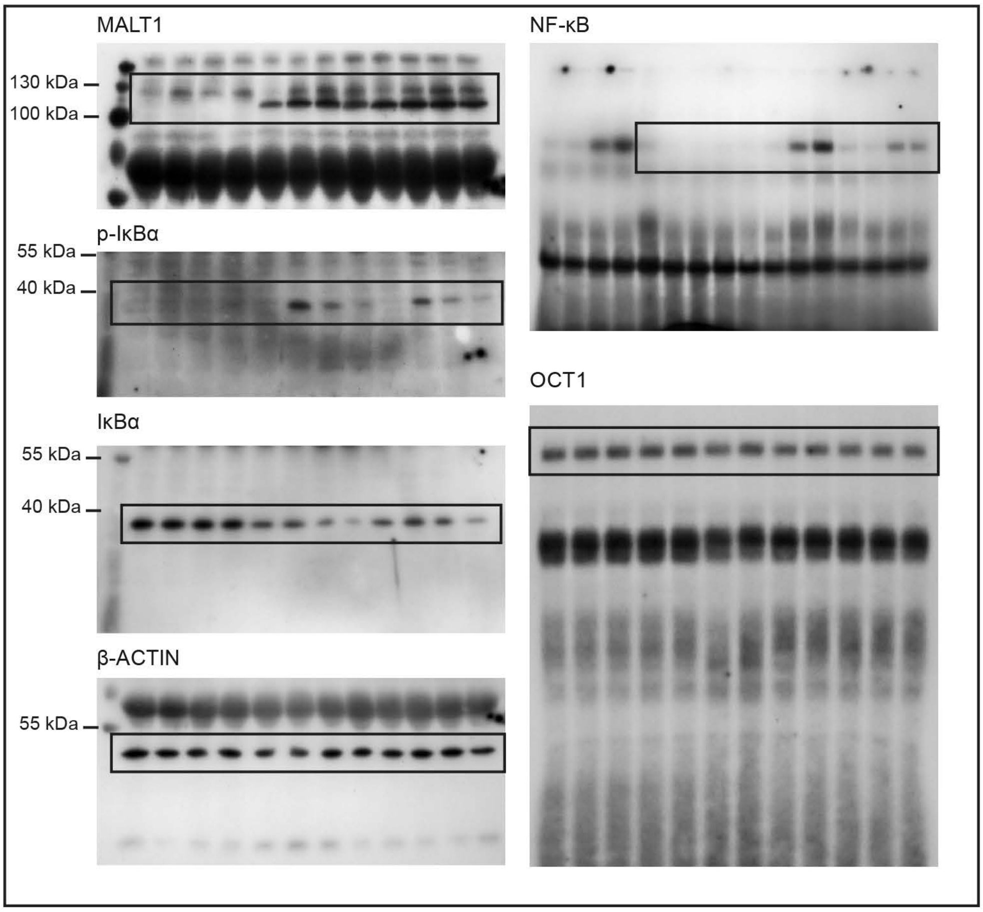

Supplementary Figure 7:

Continued.

Figure 2c

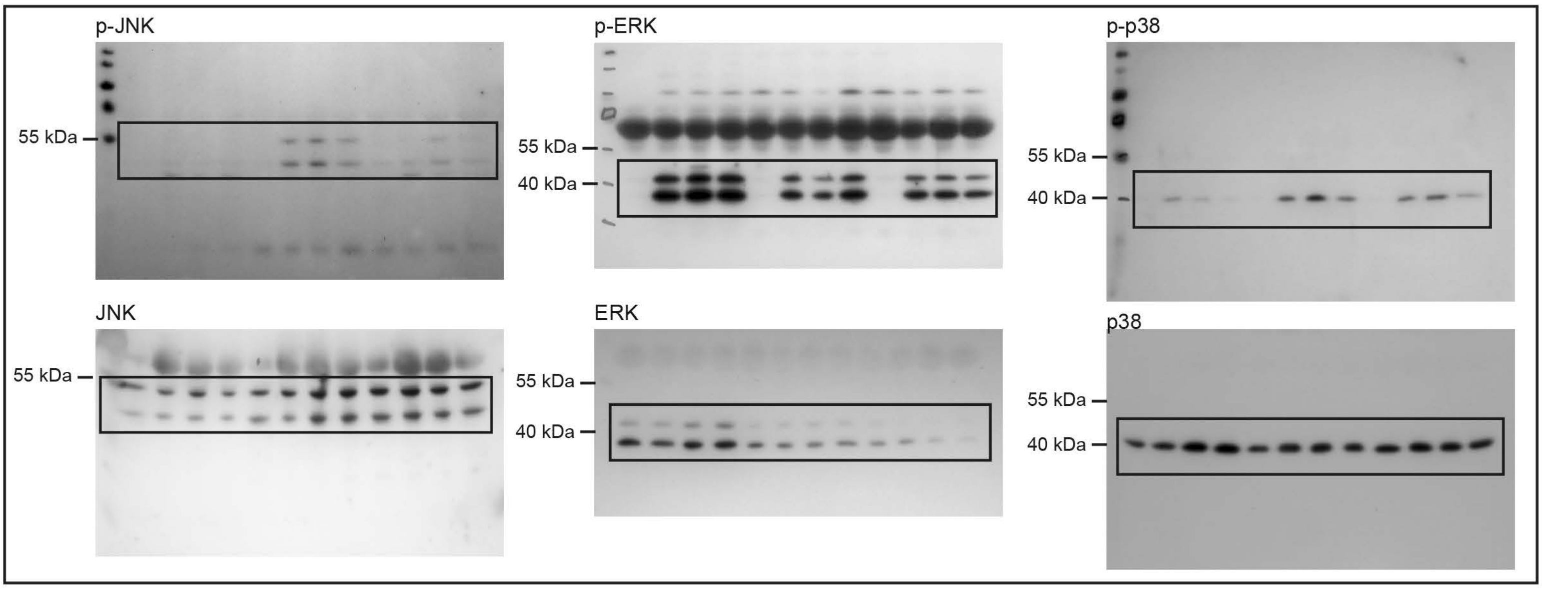

Figure 2d

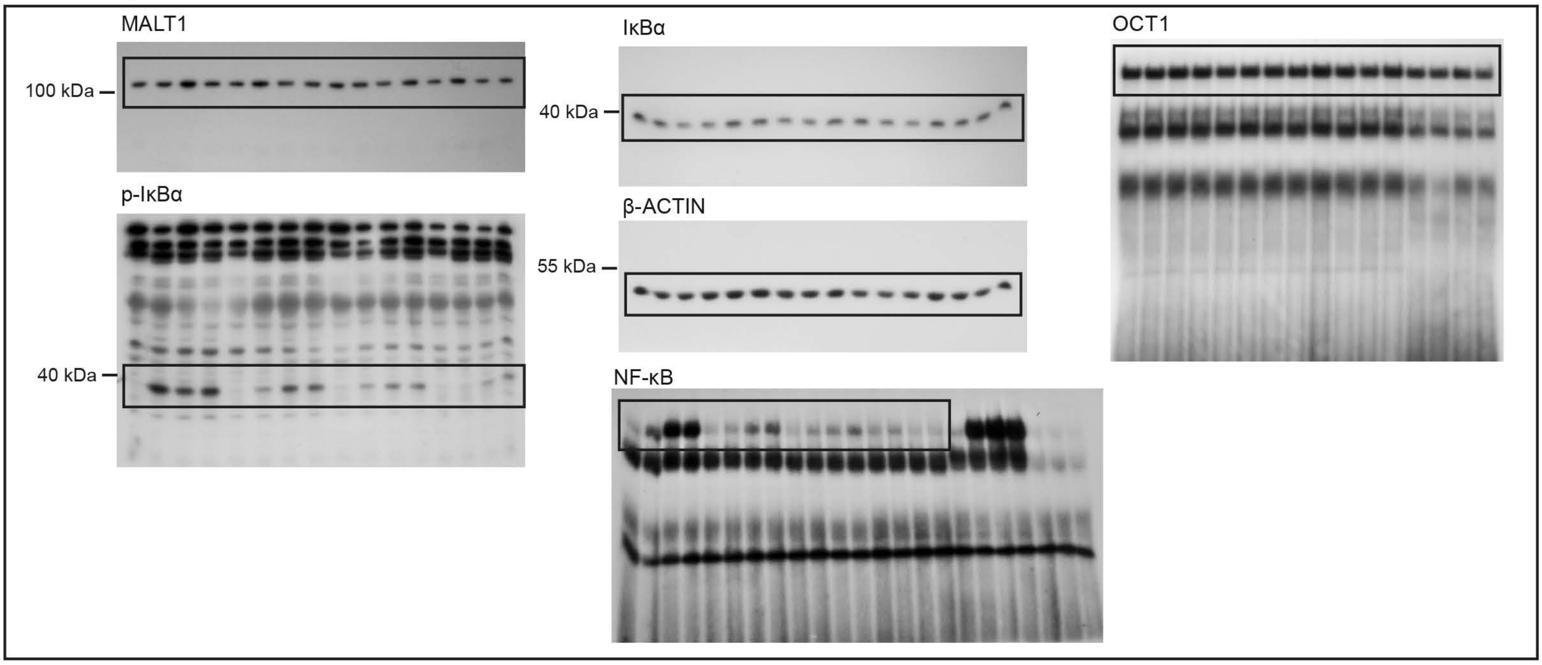

Figure 2e

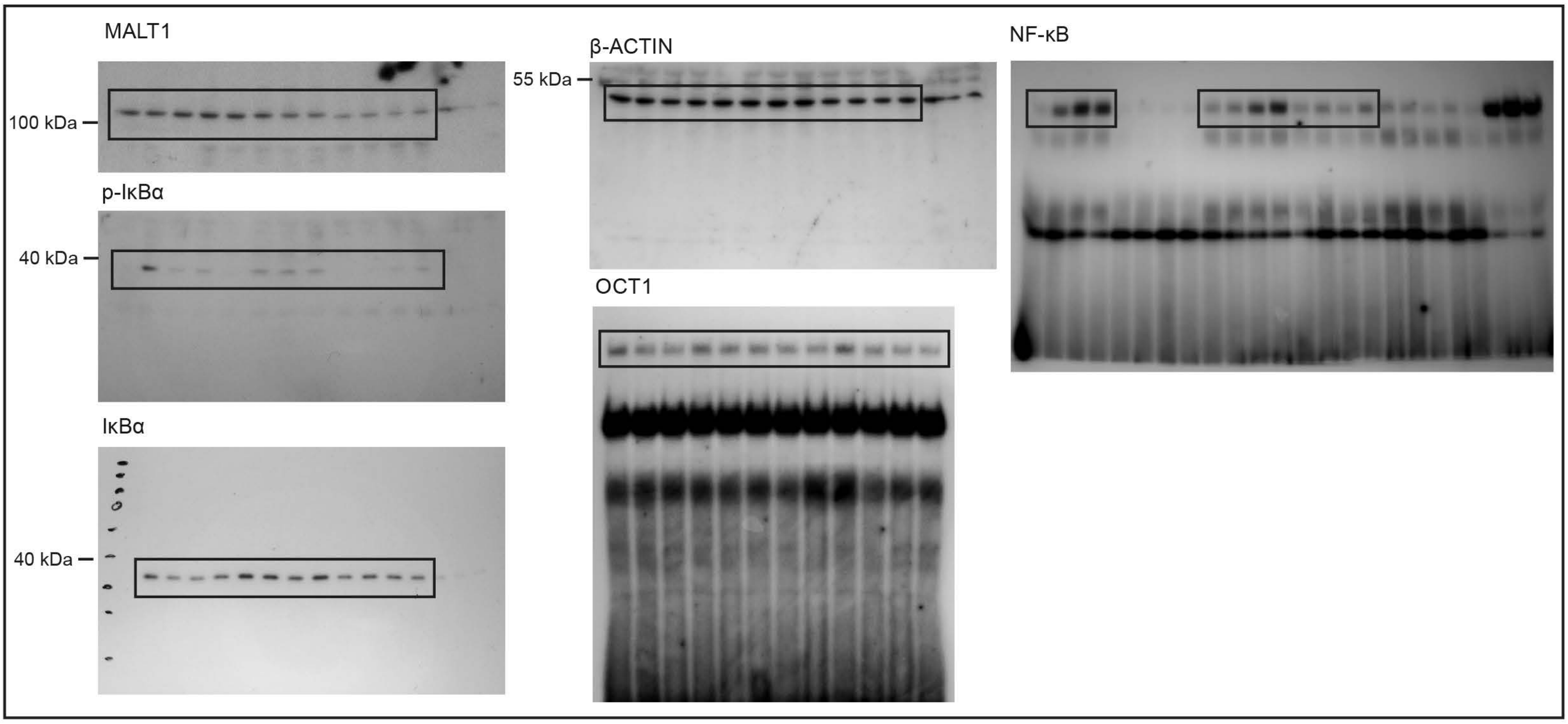

Supplementary Figure 7:

Continued.

Figure 4i

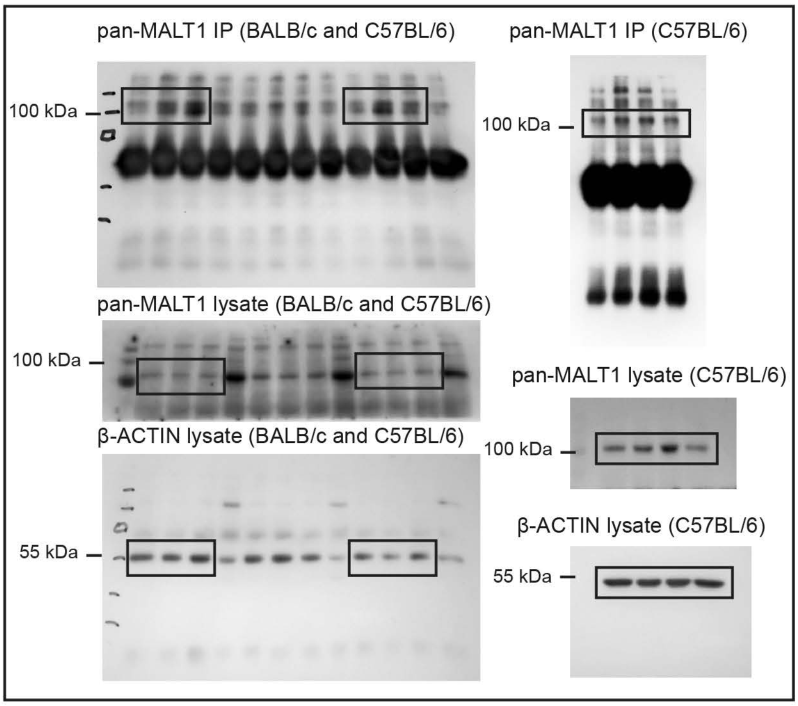

Figure 6d

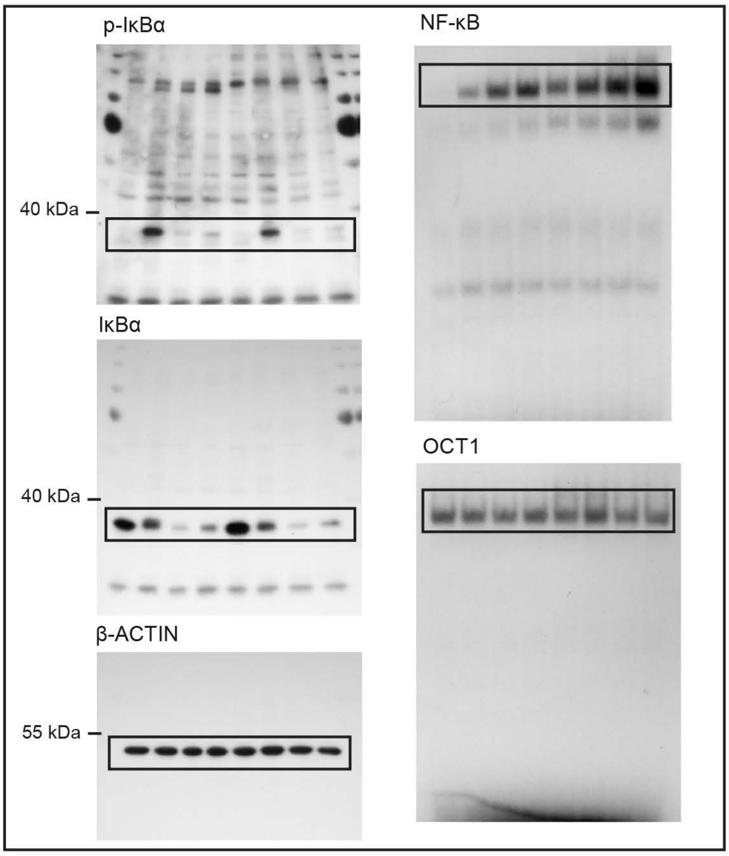

Figure 5b

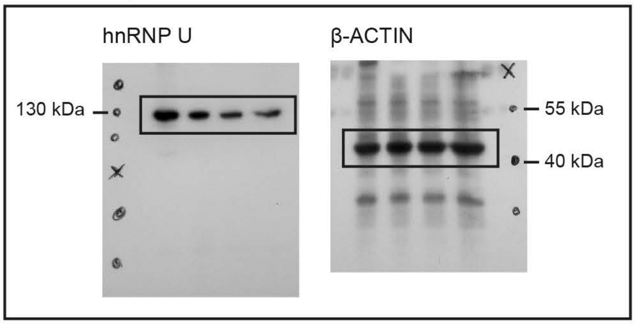

Figure 5e

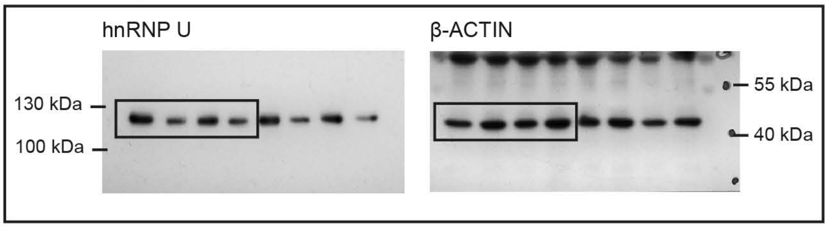

Figure 6e

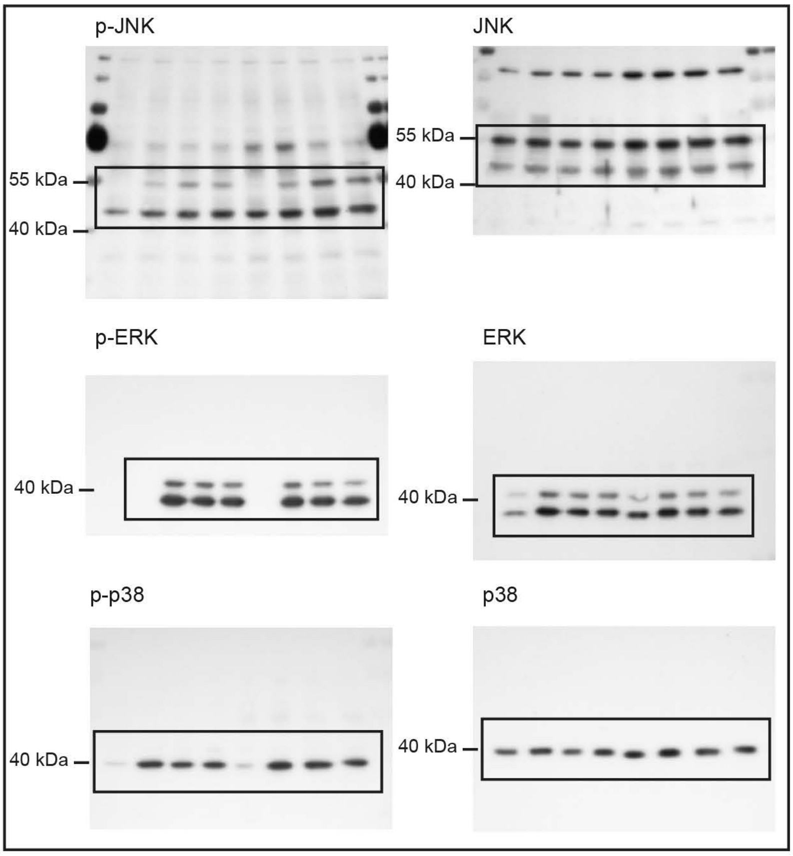

Figure 6f

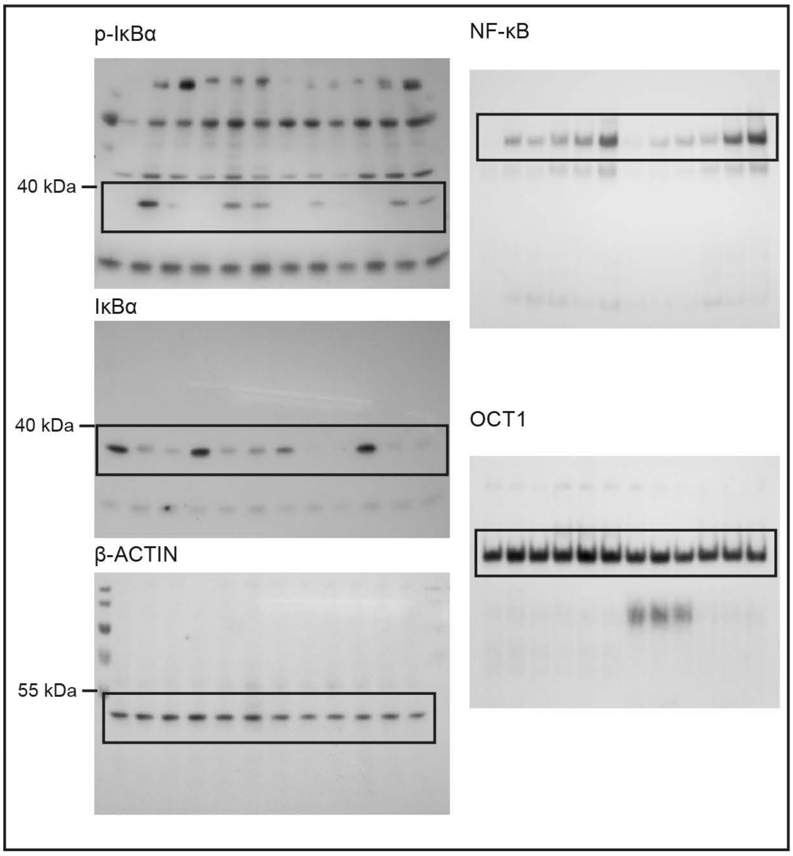

Supplementary Figure 7:

Continued.

Figure 6g

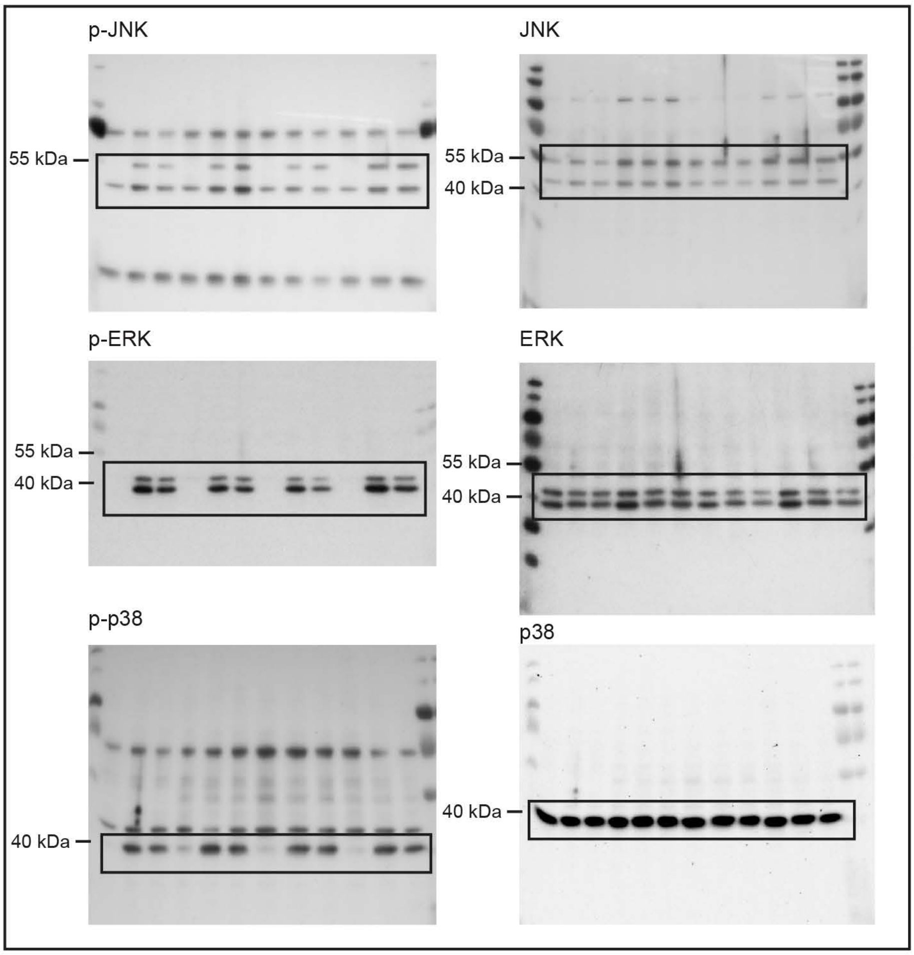

Figure 6h

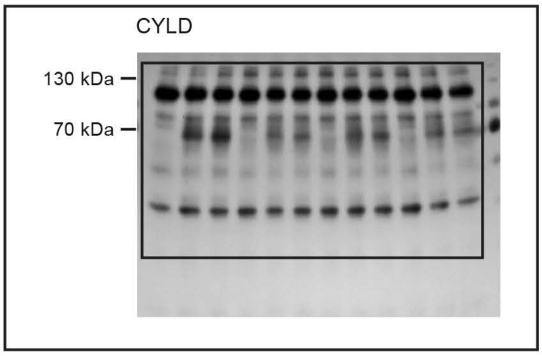

Supplementary figure 1b

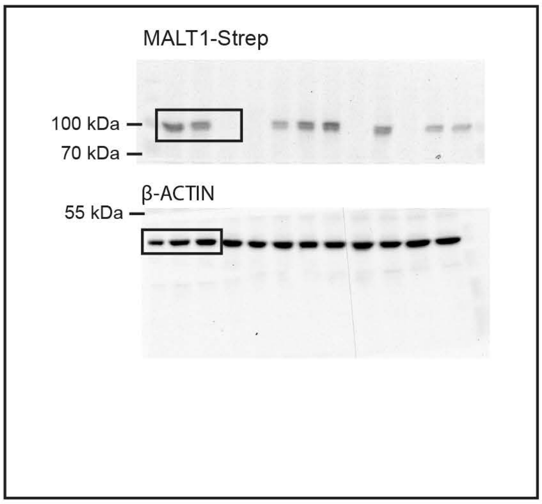

Supplementary figure 1c

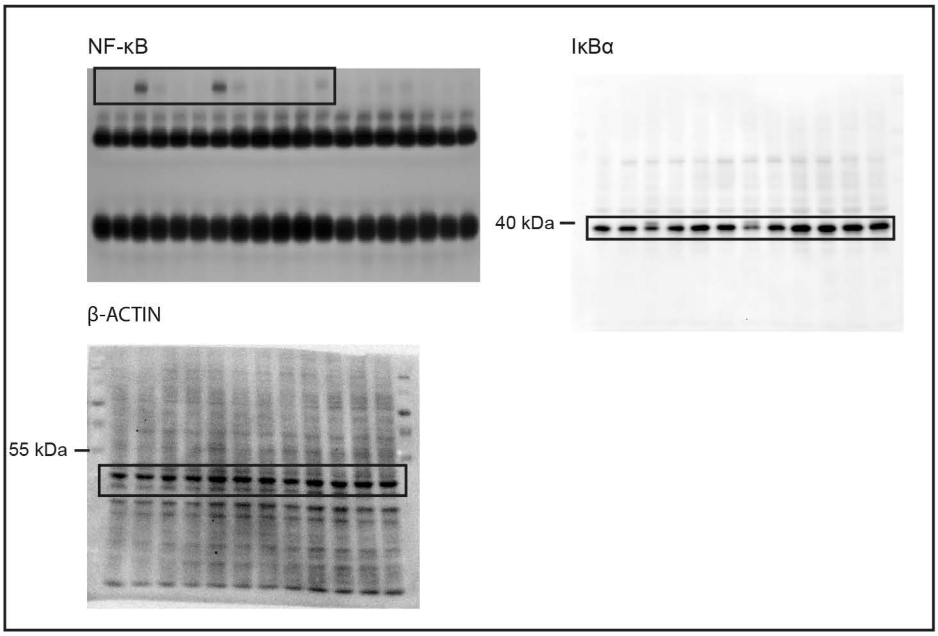

Supplementary figure 1d

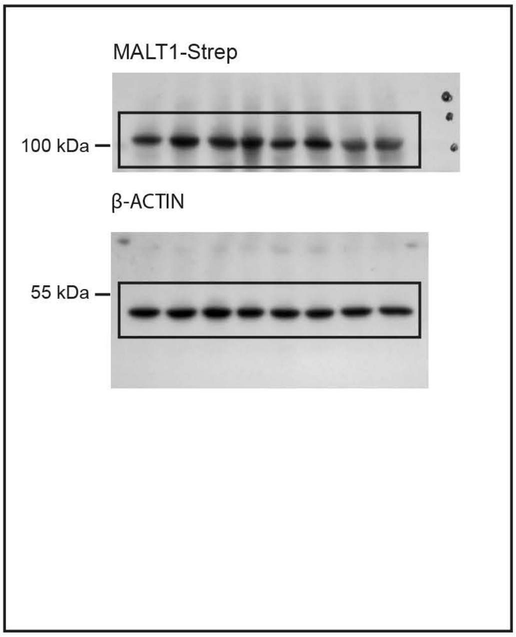

Supplementary Figure 7:

Continued.

Supplementary figure 1e

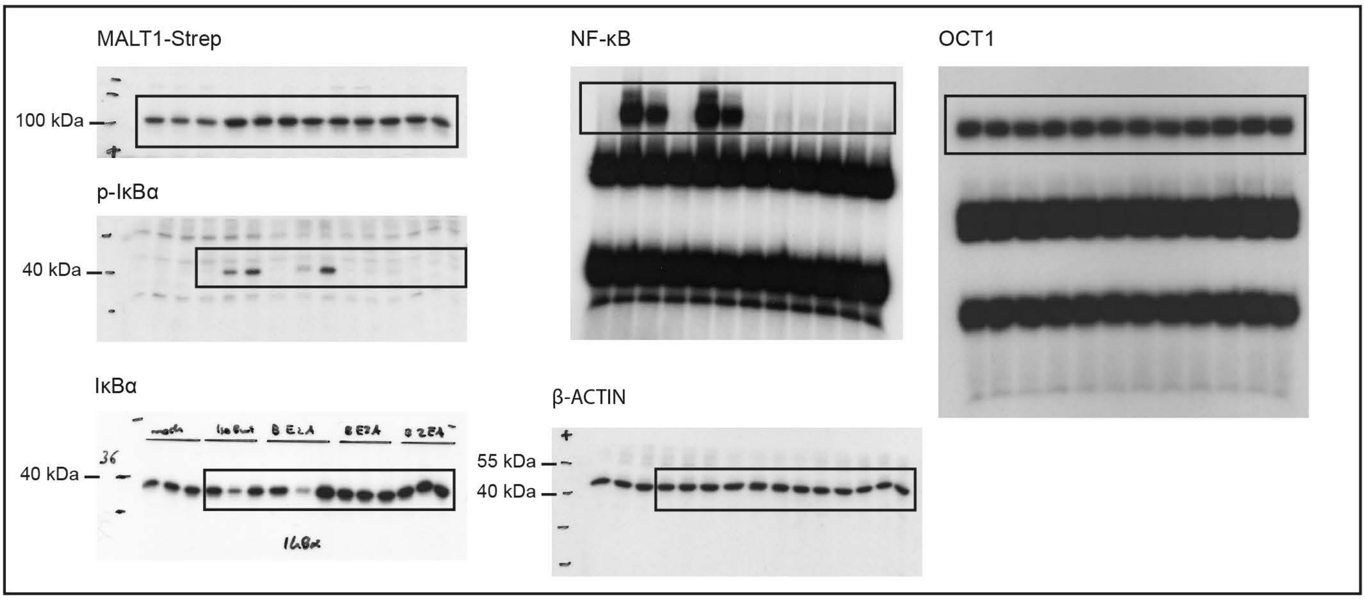

Supplementary figure 1f

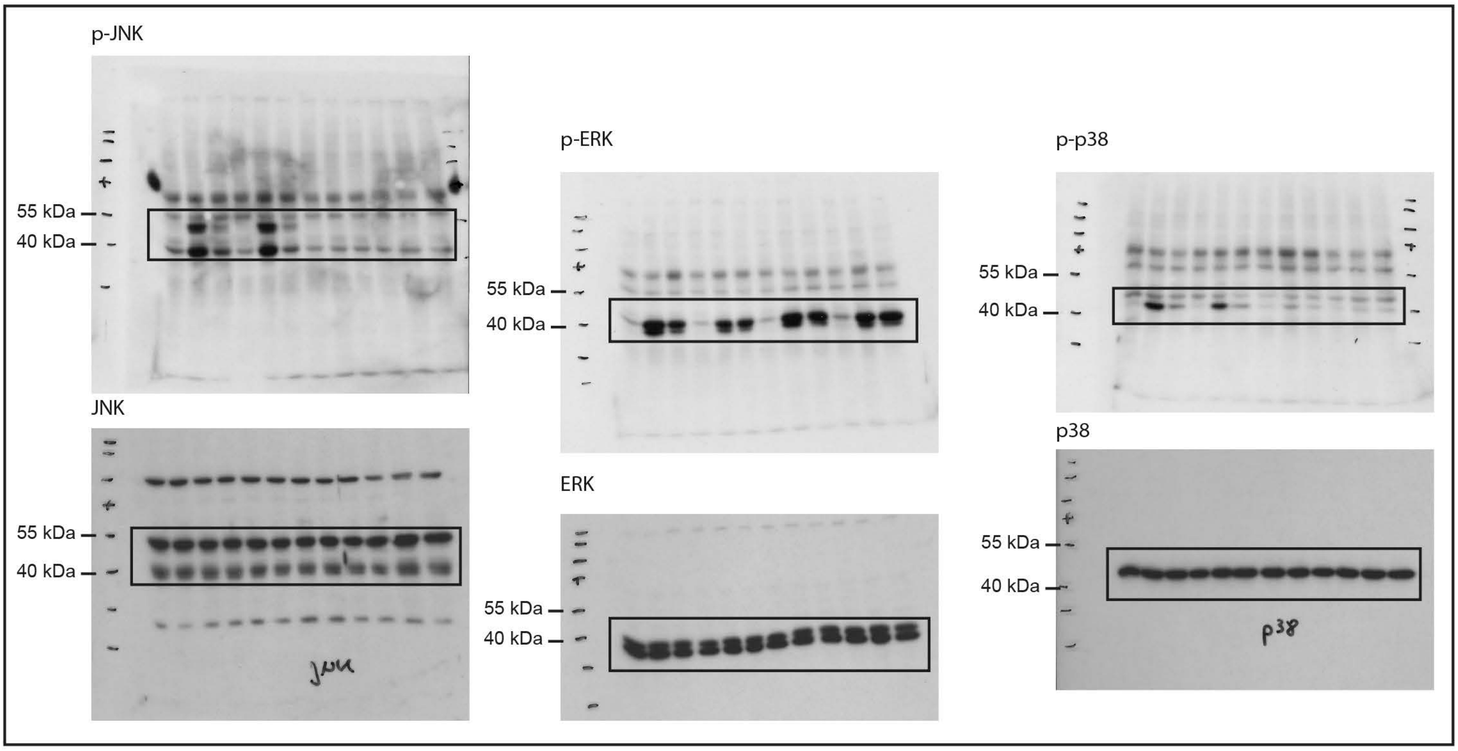

Supplementary Figure 2b

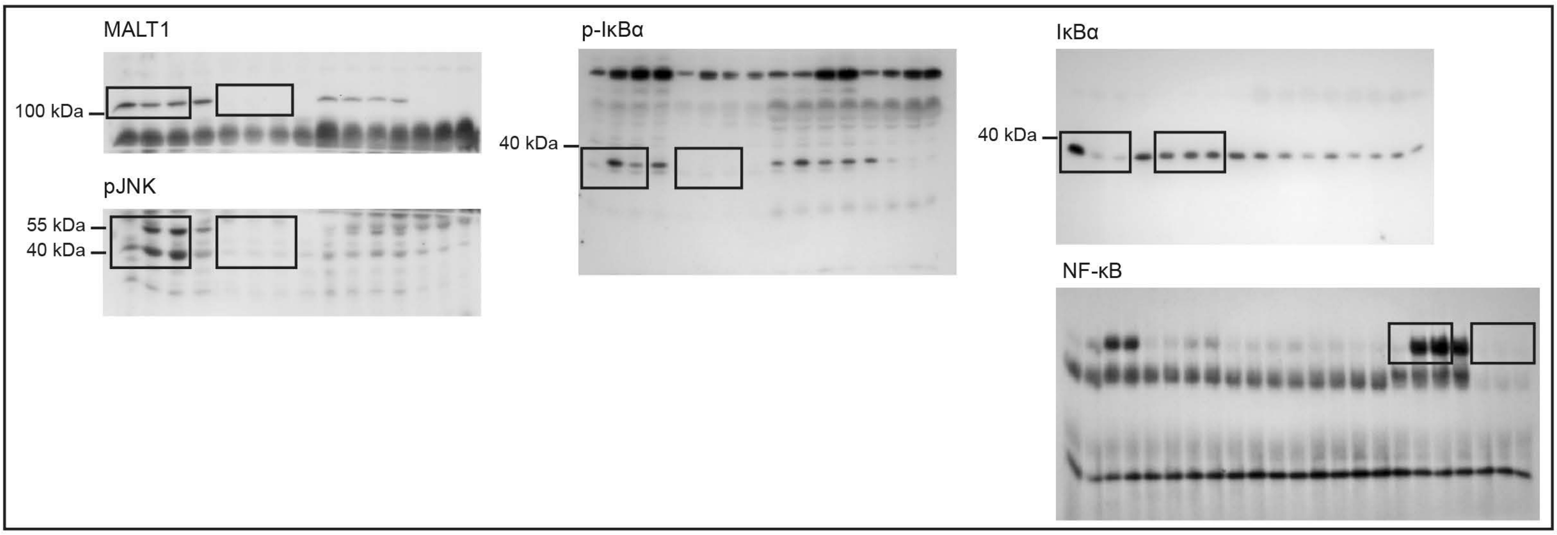

Supplementary Figure 7:

Continued.

Supplementary Figure 2c

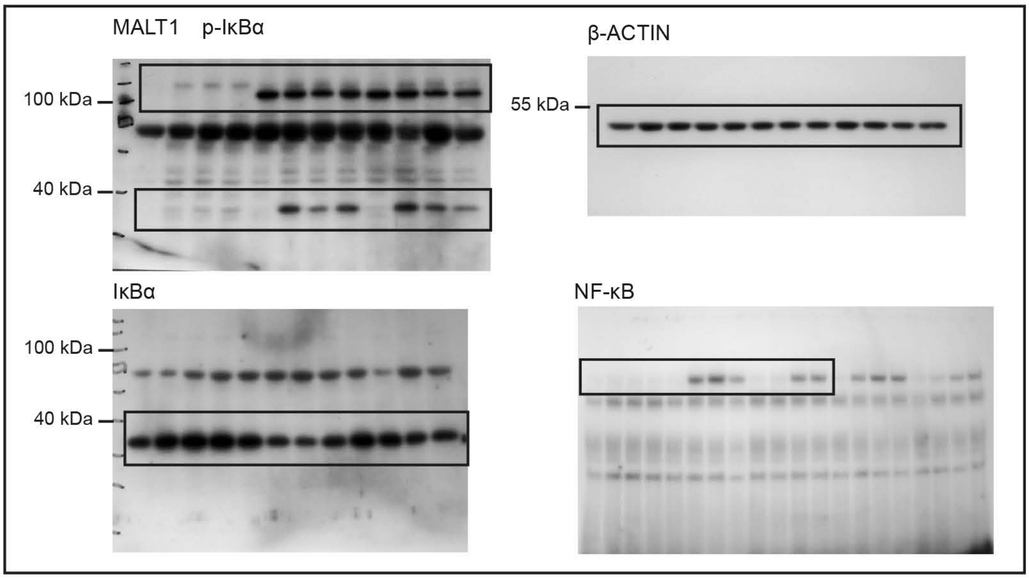

Supplementary Figure 2d

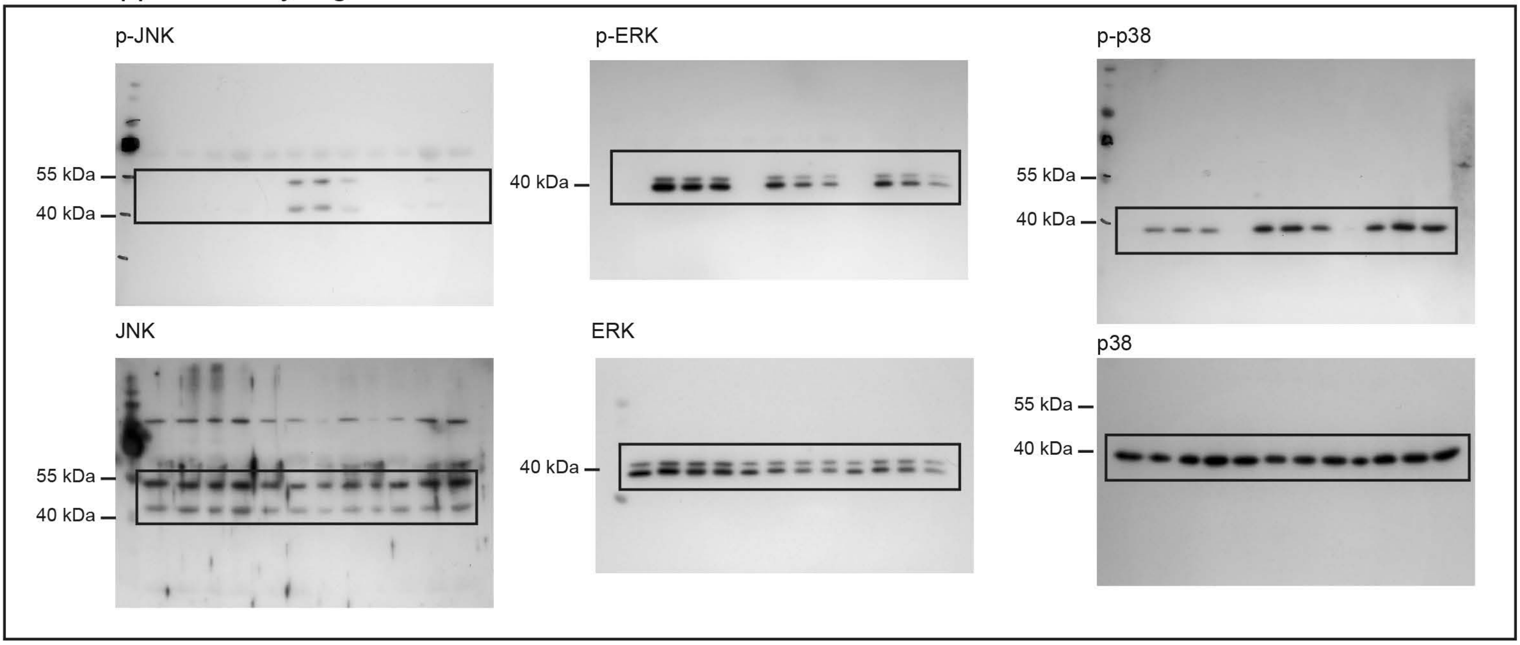

Supplementary Figure 4c

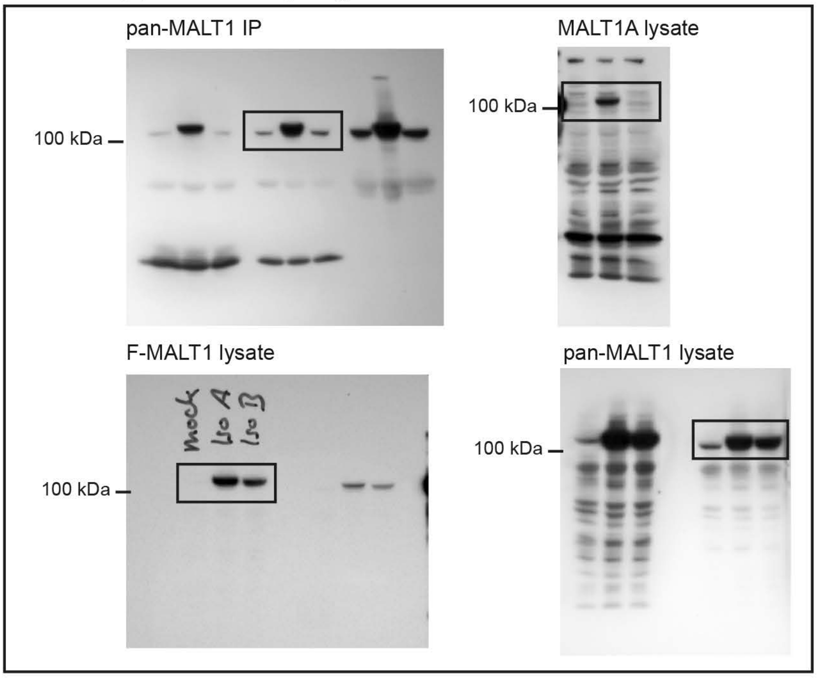

Supplementary Figure 6b

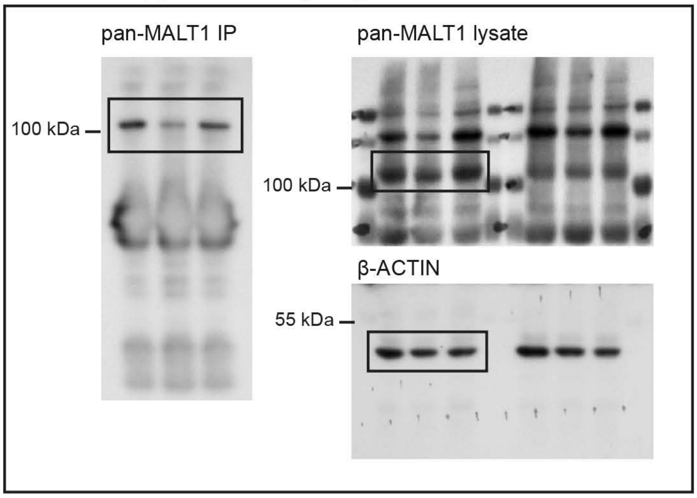

Supplementary Figure 7:

Continued.

Supplementary Figure 6d

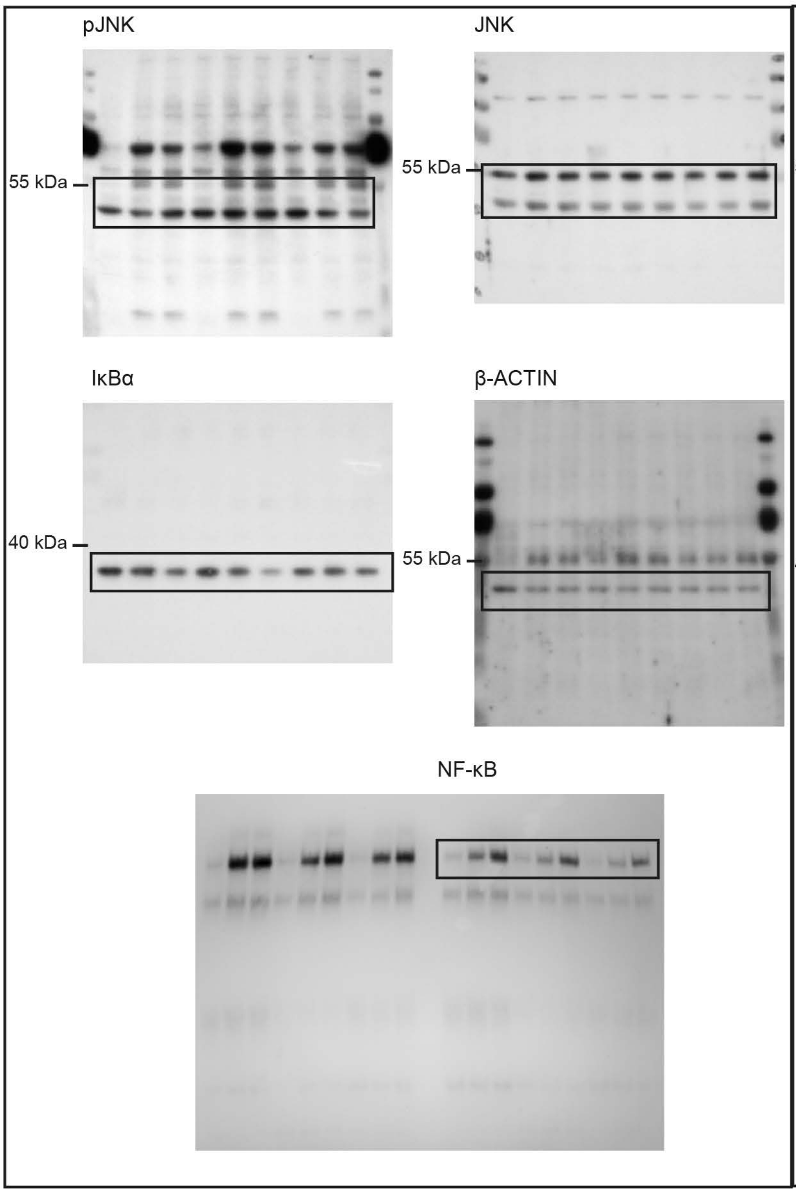

Supplementary Figure 6e

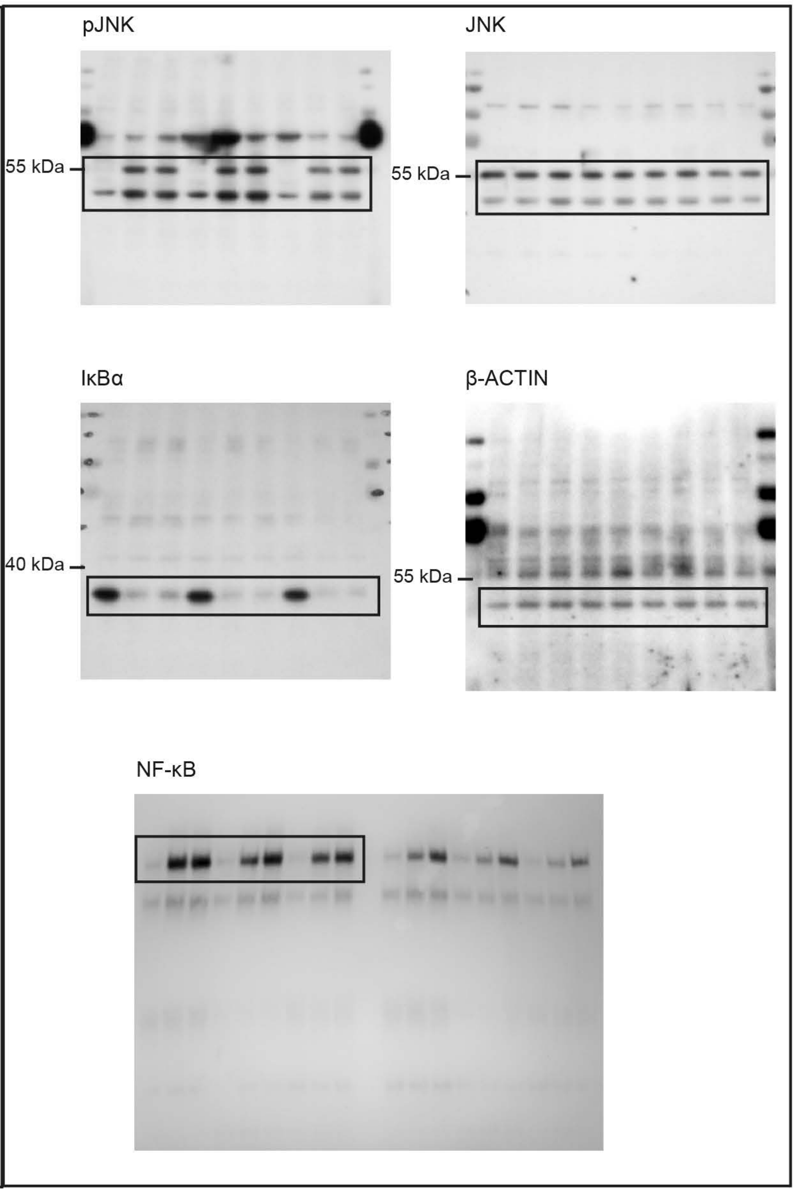

Supplementary Figure 7:

Continued.

Supplementary Table 1: RNAi to identify putative regulators of alternative MALT1 splicing

Nr.	Sample	Gene	Ratio MALT1A/MALT1B
1	con		0.32
2	con		0.25
3	A3	CELF2	0.26
4	A9	KHDRBS1	0.26
5	B3	RBM5	0.23
6	B4	RBM6	0.17
7	B5	RBMS1	0.13
8	B6	RBMS2	0.23
9	B7	SF3A1	0.30
10	B8	SF3A3	0.31
11	B10	SFRS11	0.27
12	B11	SFRS2	0.28
13	B12	SFRS7	0.23
14	C4	SRSF8	0.23
15	C5	TIA1	0.30
16	C7	CELF1	0.19
17	C8	SNRNP200	0.22
18	C10	FUS	0.28
19	C12	HNRNPK	0.28
20	D3	PTBP1	0.26
21	D4	ATXN2	0.16
22	D7	HNRNPC	0.16
23	D8	HNRNPF	0.18
24	D9	HNRNPH1	0.29
25	D10	HNRNPH2	0.15
26	D12	HNRNPL	0.18
27	E3	HNRNPU	0.65
28	E9	SFRS4	0.23
29	F6	SNRPC	0.23
30	F7	SNRPD1	0.21
31	F8	SNRPD3	0.30
32	F10	STAU1	0.18
33	G3	SF3A2	0.23
34	G5	SFRS9	0.14
35	G6	QKI	0.13
36	G7	RBM39	0.20
37	G8	RBM19	0.29
38	G12	RBM12	0.28
39	H3	RBM7	0.36
40	H4	HNRNPR	0.28
41	H5	SF3B4	0.23
42	H6	SRRM1	0.41
43	H7	KHDRBS3	0.28

44	H8	HNRNPA0	0.31
45	H9	SF3B2	0.32
46	H10	LSM6	0.31
47	H11	U2AF2	0.22
48	H12	RBM34	0.29
49	I3	RBFOX2	0.32
50	I4	SF3B1	0.23
51	I5	LSM4	0.31
52	I6	LSM14A	0.38
53	I7	STAU2	0.31
54	I8	LSM3	0.40
55	I9	RBMX	0.35
56	I10	RBM15B	0.30
57	I11	TRA2A	0.26
58	I12	SF3B14	0.24
59	J3	RBM27	0.31
60	J4	RBM47	0.31
61	J5	RBM28	0.37
62	J6	RBM22	0.36
63	J7	MBNL3	0.27
64	J8	LSM2	0.28
65	J9	PTBP2	0.28
66	J10	SF4	0.32
67	J11	RBM25	0.28
68	J12	RBM26	0.28
69	K3	ESRP2	0.24
70	K4	RBM4B	0.24
71	K5	HNRPLL	0.33
72	K6	SRSF12	0.34
73	K7	LSM14B	0.32
74	K8	RBM33	0.26
75	K9	HNRNPA3	0.23
76	K10	RBM12B	0.39
77	K11	HNRNPCL1	0.23

Supplementary Table 1:

Relative mRNA ratios of MALT1A/MALT1B after transfection of smart pool siRNA into Jurkat T cells. Quantification of radioactive PCR was done after denaturating PAGE using a PhosphoImager.

Supplementary Table 2: Murine PCR primers

Primer name	Sequence (5'-3')
MALT1 ex6 fw	ACCGAGACAGTCAAGATAGC
MALT1 ex9/10 rev	GACTTTGTCCTTTGCCAAAGG
MALT1 ex5 fw	AAGTCCTATGCCTCACTACCAGTG
MALT1 ex7/8 rev	GTTTAATTCATCTTCAGTGCACTCC
MALT1 ex6/8 rev	GATGCCCCAAATTGTTTAATTCATCTATG
MALT1 ex16 fw	GGACTCCTGAAGAAACTGGCAGC
MALT1 ex17 rev	CTTCCCCACGTTCACCTCCTGC
HMBS fw	GCGCTAACTGGTCTGTAGGG
HMBS rev	TGAGGGAAAGGCAGATATGGAGG
GAPDH fw	ACCACAGTCCATGCCATCAC
GAPDH rev	TCCACCACCCTGTTGCTGTA
IL-2 fw	GAGTGCCAATTCGATGATGAG
IL-2 rev	AGGGCTTGTTGAGATGATGC
hnRNP U fw	ATCTCGAGAGACCGTCTGAG
hnRNP U rev	CTTCACCAAGCAACATTCCAC
hnRNP D fw	TGCAACTTATCCCCAACAGG
hnRNP D rev	TCCATTCAGGAACTTGATAGAAAA
hnRNP H2 fw	CATACCTGAAGTGGATTTTCTGTC
hnRNP H2 rev	CTGACCTTCACCACGAACC
hnRNP K fw	TCTGAAGATCGGATCATTACCA
hnRNP K rev	TCTTGCATTAGAATCCTTCAACAT
hnRNP L fw	TGACTGAGGAGAACTTCTTTGAGA
hnRNP L rev	CTCTTCGAGTCCCACTCCAG
hnRNP LL fw	AGACATGATGGCTATGGATCGC
hnRNP LL rev	CACTAACCATTACAACGGAGCCA
hnRNP R fw	TCTGGAAGAGTTCAGTAAAGTCACA
hnRNP R rev	GTGCTGCTGACTTGTGATCC
SRSF3 fw	TCGTCGTCCTCGAGATGATT
SRSF3 rev	CCTATCTCTAGAAAGTGACCTGCTC
SRSF9 fw	CGAGATCGAGCTCAAGAACC
SRSF9 rev	GTAACCGTTTCTTCCATAGATCG

Supplementary Table 3: Human PCR primers

Primer name	Sequence (5'-3')
MALT1 ex6 fw (radioactive PCR)	TAATGATCGAGACAGTCAAGATAGC
MALT1 ex9/10 rev (radioactive PCR)	AACCTTGTCCTTCGCCAAAG
MALT1A fw	GAAGGTAGAAATCATCATAGGAAG
MALT1A rev	GCTTTGAGCTTGGGGTGCTCC
MALT1B fw	AAGCCCTATTCCTCACTACCAGTGG
MALT1B rev	GGATGACCAAGATTATTTAATTCATCTATG
RP2 fw	GCACCACGTCCAATGACAT
RP2 rev	GTGCGGCTGCTTCCATAA
hnRNP U fw	CGGTCCTCAAAATGAAAGGA
hnRNP U rev	CTATGGCCACCACCTCTGTT
MALT1 in1-ex2 fw	TGGAAGAAAGCTGTTGACTTGA
MALT1 in1-ex2 rev	TCCTTCAGGCTCCAGTACCTT
MALT1 in5-ex6 fw	TGTTATGTTTTGGAGCGTATTTTT
MALT1 in5-ex6 rev	CAAATCCACATAAGGCACCTA
MALT1 ex6-in6 fw	GACAGTCAAGATAGCAAGAAG
MALT1 ex6-in6 rev	CTTTGCTTAATTAAACGTTGCAG
MALT1 in6/ex7-in7 fw	TTTTCTGAAACAAGGAAGAACAGA
MALT1 in6/ex7-in7 rev	GTAGGCTAAAAGCACTCCAC
MALT1 in7 fw	AGAACCTAACTGTATTGCAAATGTATG
MALT1 in7 rev	CTTCAGTATTAGGCTCAACTCATT
MALT1 in7/ex8-in8 fw	ACACTATAACATGATTCACTGGC
MALT1 in7/ex8-in8 rev	TGTATTACTCACCAGGATGACC
MALT1 in8 fw	AAAGAAGAATGTTGTATAGGGAAAGC
MALT1 in8 rev	TGTCCACTACTGTCGGGAGAT
MALT1 ex9-in9 fw	AGCCTTTGGGTGAGTAGAAC
MALT1 ex9-in9 rev	AGCCTCCTCTTCTAAACCTTC
CD45 fw (minigene)	GGGAGCTTGGTACCACGCGTCGACC
CD45 rev (minigene)	CAGCGCTTCCAGAAGGGCTCAGAGTGG
